# The genetics of East African populations: a Nilo-Saharan component in the African genetic landscape

**DOI:** 10.1038/srep09996

**Published:** 2015-05-28

**Authors:** Begoña Dobon, Hisham Y. Hassan, Hafid Laayouni, Pierre Luisi, Isis Ricaño-Ponce, Alexandra Zhernakova, Cisca Wijmenga, Hanan Tahir, David Comas, Mihai G. Netea, Jaume Bertranpetit

**Affiliations:** 1 Institut de Biologia Evolutiva (UPF-CSIC), Departament de Ciències Experimentals i de la Salut, Universitat Pompeu Fabra, Barcelona, Catalonia, Spain; 2 College of Medical Laboratory Sciences, University of Science and Technology, Omdurman, Sudan; 3 Banoon ART and Cytogenetics Centre, Bahrain Defence Force Hospital, Manama, Kingdom of Bahrain; 4 Departament de Genètica i de Microbiologia, Grup de Biologia Evolutiva (GBE), Universitat Autonòma de Barcelona, Bellaterra (Barcelona), Spain; 5 University of Groningen, University Medical Center Groningen, Department of Genetics, Groningen, The Netherlands; 6 Sudan Medical and Scientific Research Institute, University of Medical Sciences and Technology, Khartoum, Sudan; 7 Department of Internal Medicine and; 8 Radboud Center for Infectious Diseases, Radboud University Nijmegen Medical Centre, Nijmegen, The Netherlands

## Abstract

East Africa is a strategic region to study human genetic diversity due to the presence of ethnically, linguistically, and geographically diverse populations. Here, we provide new insight into the genetic history of populations living in the Sudanese region of East Africa by analysing nine ethnic groups belonging to three African linguistic families: Niger-Kordofanian, Nilo-Saharan and Afro-Asiatic. A total of 500 individuals were genotyped for 200,000 single-nucleotide polymorphisms. Principal component analysis, clustering analysis using ADMIXTURE, F_ST_ statistics, and the three-population test were used to investigate the underlying genetic structure and ancestry of the different ethno-linguistic groups. Our analyses revealed a genetic component for Sudanese Nilo-Saharan speaking groups (Darfurians and part of Nuba populations) related to Nilotes of South Sudan, but not to other Sudanese populations or other sub-Saharan populations. Populations inhabiting the North of the region showed close genetic affinities with North Africa, with a component that could be remnant of North Africans before the migrations of Arabs from Arabia. In addition, we found very low genetic distances between populations in genes important for anti-malarial and anti-bacterial host defence, suggesting similar selective pressures on these genes and stressing the importance of considering functional pathways to understand the evolutionary history of populations.

East Africa is a strategic region to study human genetic diversity due to the presence of ethnically, linguistically, and geographically diverse populations. North African and sub-Saharan African populations in East Africa are connected by the Nile River. It has been proposed that the Nile River Valley acted during history as a genetic corridor allowing gene flow between North and South in Eastern Africa^1^,^2^, two very distinct regions in terms of human populations. Both the possibility that East Africa is the place of origin of modern humans, and the introduction of a North African genetic component into the sub-Saharan Africa, might have contributed to East Africa having the greatest level of regional substructure in the continent and the world.

East Africa complexity can be seen in the fact that all families of continental African languages are represented in the region. Continental African languages have been classified into four major language families: Afro-Asiatic, Nilo-Saharan, Niger-Kordofanian (or Niger-Congo), and Khoisan. Afro-Asiatic, spoken predominantly by northern and eastern African pastoralists and agro-pastoralists, covering North Africa, includes the Semitic, Cushitic, and ancient Egyptian (Coptic) languages. Nilo-Saharan, spoken predominantly by eastern and central African pastoralists, includes in its main Chari-Nile branch the Central Sudanic and Eastern Sudanic (also called Nilotic) languages. Niger-Kordofanian, spoken predominantly by agriculturalist populations across western, eastern, central, and southern Africa, includes the Bantu languages^3^,^4^. It is interesting to note that the outlier Kordofanian branch, that expanded the previous Niger-Congo family, is represented in the present study.

In an extensive and detailed study, Tishkoff *et al.*^5^ characterized the population substructure in Africa and identified 14 ancestral components predominantly associated with linguistic affiliations. Recent studies have further analysed these ancestral components to explain their origins^6^,^7^. Despite the genetic and linguistic complexity present in East Africa, there are some populations that have not been properly assessed and which might provide a complementary understanding of the population diversity in the region.

Here, we focus on the region of Sudan and South Sudan with some other external related populations (Ethiopians in the East; Fulani, in the West); we refer as Sudanese Region to the ensemble (see Fig. 1 and Table 1). The genetic population history of the Sudan has been interrogated using non-recombinant markers (mitochondrial DNA, Y-chromosome)^1^,^8^ and a small number of autosomal markers^9^. More recent studies have analysed a significant number of microsatellites^5^,^10^ and of single nucleotide polymorphisms (SNPs)^6^,^11^, with results suggesting that African populations may have maintained a large and subdivided population structure throughout most of their evolutionary history^5^. But as the Sudanese region is inhabited by ethnically, linguistically and culturally diverse populations, studies using a larger number of markers and representative samples of the ethno-linguistic groups of the area are needed for fine-scale population structure inference.

The first aim of this study was to provide new insights into the genetic history of East African populations by analysing six Sudanese ethnic groups belonging to the main African linguistic families spoken in the region (Afro-Asiatic, Nilo-Saharan and Niger-Kordofanian), in addition to ethno-linguistic neighbouring groups (Nilotes of South Sudan, nomadic Fulani from the Sahel, and Ethiopians). We assessed the genetic diversity and relationships between these different ethno-linguistic groups to clarify the genetic history of East Africa. The second aim of the study was to use genetic distance estimated as F_ST_ to identify putative signals of adaptive selection in these populations, with a focus on immunological adaptation in anti-malarial, anti-bacterial and anti-fungal defence genes.

## Results

### Population Structure

We applied a principal component analysis (PCA) to investigate the population structure of the new populations genotyped in this study from the Sudanese region (Supplementary Fig. S1a). PC1 (3.56% of the variation) follows a North-South cline and separates populations inhabiting the region between the Nile River and the Red Sea (Nubians and Arabs along the Nile, Beja and Ethiopians along the coast) from Darfurians and Nuba of South-West Sudan, and Nilotes of South Sudan. Copts are a separated group close to the North-East populations, in a more outlier position: they are the extreme of the northern genetic component. PC2 (0.7%) separates the nomadic Fulani from the other populations.

Next, we combined our new populations (140 K data set) with previously studied populations of special interest for this analysis: Qatar^12^, Egypt^13^, and three sub-Saharan populations (Luhya, Yoruba and Maasai) from 1000 Genomes Project^14^ to have external references both in the north and south of the Sudanese region. This new data set contains 14,343 SNPs (14 K data set). Even if the number of SNPs in this second set is small, it is enough to differentiate components in the African genetic landscape^15^. Fig. 2 shows a PCA of this extended data set, where East African populations are distinct from both sub-Saharan and North African populations. PC1 (6.08%) separates between populations from North Africa/Middle East and sub-Saharan Africa (Fig. 2a). Copts are closer to North African and Middle East populations but remain as a separate cluster when PC2 is considered. PC2 (1.46%) along with PC1 separate the two homogeneous clusters of North-East and South-West populations: Nubians, Arabs, Beja and Ethiopians on one hand, and Nuba, Darfurians and Nilotes on the other. PC2 separates all Sudanese and Ethiopian populations from the rest. PC3 (0.56%) differentiates West-African populations (Fulani and Yoruba) from Sub-Saharan East African populations (Maasai) (Fig. 2b). Both PC analysis using data sets with different number of SNPs preserve the topology of the populations. As expected, with a low number of SNPs we observe a higher intra-population variation (Supplementary Fig. S1b).

To test whether these particular sets of Immunochip SNPs (140 K and 14 K data sets) can recover population structure, we extracted 1000 Genome data from world-wide populations and observed that the genetic structure between them is maintained across the different data sets of SNPs used (Supplementary Fig. S2). In addition, the effect of ascertainment bias in the Immunochip was also assessed using a subset of presumably neutral SNPs (SNPs located in intergenic regions) (Supplementary Fig. S3). No strong effect of ascertainment bias was observed. Thus, our inferences of population structure seem robust to the sample size and particularities of the data sets of SNPs used.

Pairwise F_ST_ statistic, a measure of global population differentiation, confirmed the PCA clustering (Supplementary Table S2, Supplementary Fig. S5). Populations geographically close had low average F_ST_ values, even though population-specific characteristics were emphasized by excluding population outliers (Supplementary Fig. S4). The lowest average F_ST_ (0.003) was found both in the pair Arabs and Nubians, located at the Nile River Valley, and in the pair Beja and Ethiopians, located at the coast. Among North-East populations, Nubians had the highest F_ST_ values when compared with Beja and Ethiopians (average F_ST_ of 0.006 and 0.007 respectively). South-West populations showed higher population differentiation among themselves than North-East populations. When comparing North-East populations with South-West populations, all comparisons have a high F_ST_ (between 0.044 and 0.054). Copts, with a strong individual heterogeneity, are more similar to Arabs (F_ST_ = 0.019) than to any other East African population. Copts and South-West populations are the most distant populations (F_ST_ > 0.1). Fulani had on average lower F_ST_ values when compared to South-West (Nuba, Darfurians and Nilotes) than to North-East populations (Nubians, Arabs, Beja and Ethiopians). These values show a complex situation beyond the simple North African versus Sub-Saharan Africa main differentiation.

To test the hypothesis that geographically close populations are genetically similar, we performed a Mantel test to determine to which extent geographic and genetic distances (as pairwise F_ST_) between populations are correlated. We found a significant positive correlation between genetic and geographic distance (r = 0.5105, p-value < 0.0001).

### Population Admixture

To infer the ancestral populations of the East African individuals, we run ADMIXTURE from *k =* 2 to *k =* 10 in the 14 populations (the analysis for the internal nine populations is presented in Supplementary Fig. S7, S10). We analysed the results from *k =* 2 to *k =* 5 as higher numbers of ancestral components do not have a clear origin. A complex pattern of admixture is observed in East African populations (Fig. 3). At *k =* 2, we already detect different ancestries in the Sudanese populations. Copts show a common ancestry with North African and Middle Eastern populations (dark blue), whereas the South-West cluster (Darfurians, Nuba and Nilotes) share an ancestry component (light blue) with sub–Saharan samples. The North-East cluster (Beja, Ethiopians, Arabs and Nubians) shows both components, although the main component (~70%) is that detected in North Africa and Middle East (Fig. 3).

At *k =* 3 (best statistically supported model, see Supplementary Fig. S8b), a new component (light green) appears, well differentiated from other South Saharan or North Africa and Middle East populations. This component defines South-West Sudanese populations (Nuba and Darfurians) and Nilotes of South Sudan and is different from the main sub-Saharan component as seen in Yoruba and Luhya. This Nilo-Saharan component, which is also found at lower percentage in the North-East cluster and Maasai, will be outlined in the discussion.

Copts share the same main ancestral component than North African and Middle East populations (dark blue), supporting a common origin with Egypt (or other North African/Middle Eastern populations). They are known to be the most ancient population of Egypt and at *k* = 4 (Fig.3), they show their own component (dark green) different from the current Egyptian population which is closer to the Arabic population of Qatar.

It is noteworthy the case of the Fulani, which feature more Sudanese ancestry (>45%) than North African (<40%) or sub–Saharan (<15%) and at *k* = 5 show their own component (Fig.3). They have a high individual component variance suggesting a recent admixture event in this population.

To formally test the results of the admixture analysis, we applied the three-population test (f_3_ statistics)^16^. We used all possible pairs of populations as surrogates of the ancestral populations of each ethno-linguistic group. All populations that have a complex pattern of admixture (Fig. 3) showed statistically significant results (Z-score <−4, p-value <3.2 × 10^−5^): those of the North-East cluster (Beja, Ethiopians, Arabs and Nubians) and Fulani. Populations from the North-East cluster: Beja, Ethiopians, Arabs and Nubians (Table 2) may be explained as admixture products of an ancestral North African population (similar to Copts) and an ancestral South-West population (Nuba, even if in one case Darfurians have better fit). These four populations had an intermediate position between Copts and South-West Sudanese populations both in the PC and admixture analyses.

Fulani, who are known to have West-African ancestry, have a negative f_3_ with Copts and Yoruba as source populations (Table 2). As they have a complex history and present high levels of admixture with different populations and high individual variance, this three-population phylogeny seems naïve to explain their complex population history. None of the South-West populations (Darfurians, Nuba and Nilotes) appear as admixed in the three-population test. This result fits the ADMIXTURE analysis (Fig. 3 and Supplementary Fig. S10) and it confirms a specific ancestral component for these populations.

### Low genetic distance between populations for genes involved in infectious diseases

We studied the effects of infectious pressures on the genetic make-up of populations in East Africa by calculating genetic distances (as F_ST_) between populations using the genetic variation in genes involved in defence against different agents. We selected among the genes genotyped in the Immunochip those associated with resistance/susceptibility to malaria^17^ (Supplementary Table S5), those related to host defence against bacteria^18^ (Supplementary Table S6), and those related to host defence against fungi (Supplementary Table S7). For every pair of populations, the mean F_ST_ of those genes was compared to the mean F_ST_ of a set of randomly selected SNPs from genic regions with the same sample size and similar MAF, using a permutation test (10.000 permutations). All pairwise comparisons showed that the mean F_ST_ score of malaria-related genes was significantly lower than the mean F_ST_ score of the sampling distribution (Fig. 4). This suggests that all these populations have suffered a strong selective pressure in the same direction in genes related to malaria resistance. In the case of antibacterial host defence genes, all comparisons except Copts and the North-East populations had a mean F_ST_ score significantly lower than the sampling distribution mean (Fig. 5). For the genes encoding proteins important for antifungal defence only three comparisons showed populations with a mean F_ST_ score lower than the sampling distribution: Copts compared to South-West populations, Copts compared to Fulani, and North-East populations compared to South-West (Fig. 6).

We tested whether the specific SNPs present in the Immunochip for genes related to infectious diseases are a representative sample of all the SNPs of those genes using 1000 Genomes data of African populations (Supplementary Table S8). Results show that the SNPs present in the Immunochip for the genes of interest can be considered as a representative sample of all the SNPs in those genes.

## Discussion

In this study we present an extensive genome-wide data set characterizing East African human genetic diversity in populations from Sudan, South Sudan and Ethiopia. We further analyse the Nilo-Saharan ancestral component within the variation of South-Saharan Africans. This component belongs linguistically to Eastern Sudanic languages and geographically to South and West of Sudan and South Sudan, including highly diverse ethnic groups in a similar genetic background. This component was identified in previous studies using Nilotic populations, but it was not analysed in other Nilo-Saharan populations, such as Darfurians or the Nuba people. In addition, we show convergent evolutionary pressures exerted on genes involved in anti-malaria and anti-bacterial host defence processes.

Africa genetic landscape is shaped by geographic barriers^19^, but the forces clustering populations vary depending on the scale. On a regional scale, East Africa populations cluster mainly by linguistic affiliation^5^. However, it has been previously reported that language plays a lesser role in the genetic clustering of Sudanese populations, as geography is the main factor that groups them^10^. This observation is supported by our data, as shown in the PCA (Fig. 2.), where PC1 represents a north-east to south-west axis delimited by the Nile River and its main tributaries: the Blue Nile and the White Nile. Genetic and geographic distances between populations of the Sudanese region are positively correlated (Mantel test; r = 0.5105, p-value < 0.0001), with Sudanese populations clustering in four groups according to their geographic location (Supplementary Fig. S1).

Nubians are the only Nilo-Saharan speaking group that does not cluster with groups of the same linguistic affiliation, but with Sudanese Afro-Asiatic speaking groups (Arabs and Beja) and Afro-Asiatic Ethiopians (Supplementary Fig. S1a). Y-chromosome and mitochondrial DNA studies reported Nubians to be more similar to Egyptians than to other Nilo-Saharan populations^1^,^8^: Nubians were influenced by Arabs as a direct result of the penetration of large numbers of Arabs into the Nile Valley over long periods of time following the arrival of Islam around 651 A.D^20^.

Interestingly, our analyses shows a unique ancestry for Sudanese Nilo-Saharan speaking groups (Darfurians and Nuba) related to Nilotes of South Sudan, but not to other Sudanese populations or sub-Saharan populations (Fig. 3). This ancestral component is not present in places where the Bantu expansion left a strong footprint and creates a different genetic background that is not found among most African populations. Tishkoff *et al.*^5^. reported a common ancestry of Nilo-Saharan speaking populations. We also found this relationship of Nilo-Saharan Sudanese populations with other Nilo-Saharan populations from Kenya (Maasai), but not as strong, as Maasai show their own genetic component at *k =* 6, which is different from the Sudanese component (Supplementary Fig. S7) and do not cluster with our Nilo-Saharan speaking populations. In a previous Y-chromosome study^8^, most Nilo-Saharan speaking populations, except Nubians, showed little evidence of gene flow with other Sudanese populations.

The presence of the core of Nilo-Saharan languages in the confluence of the two Nile rivers suggests that the Sudanese region is the place of origin of the Nilo-Saharan linguistic family despite their fragmented distribution, as shown by the location of the Nubian language^21^,^22^. It is interesting to note that Nuba populations constitute an homogeneous group, even if some speak Kordofanian (of the Niger-Kordofanian family) and others different languages of two branches of the Nilo-Saharan family. Their genetic composition denotes their Nilo-Saharan origin, with linguistic replacements in some groups.

Population displacement, whether it is followed with cultural or genetic exchange with local populations, would explain why not every Nilo-Saharan speaking group has this genetic component (as is the case of Nubians) and not every population that has it is mainly formed by Nilo-Saharan speakers (as is the case of Niger-Kordofanian speaking Nuba).

The North African/Middle Eastern genetic component is identified especially in Copts. The Coptic population present in Sudan is an example of a recent migration from Egypt over the past two centuries. They are close to Egyptians in the PCA, but remain a differentiated cluster, showing their own component at *k* = 4 (Fig. 3). Copts lack the influence found in Egyptians from Qatar, an Arabic population. It may suggest that Copts have a genetic composition that could resemble the ancestral Egyptian population, without the present strong Arab influence.

A population that shows signals of recent admixture is the Fulani. Fulani are nomadic pastoralists who speak a Niger-Kordofanian (Niger-Congo) language and occupy a large area in Africa’s Sahel. Their origin is still controversial, as mitochondrial DNA indicates a West African and traces of North African origin^23^, whereas Y-chromosome studies showed shared ancestry with Afro-Asiatic and Nilo-Saharan Sudanese populations^8^. This shared ancestry with East African populations can be seen in Fig. 3 (*k* = 3), suggesting that they have admixed with local populations. This finding does not agree with studies of Fulani people in the Lake Chad Basin which reported that Fulani from West Africa’s Sahel usually have consanguineous marriages and do not seem to have admixed with local farmers^24^. These data together suggest differentiated genetic legacy in different Fulani populations from various geographic regions of the continent.

The second objective of our study was to analyse how infectious pressures affected the genetic variation of East African populations. The *a-priori* hypothesis was that selective pressures on host defence genes induced by similar infections would determine lower genetic distances between populations, as compared with a genome-wide distribution: a *de facto* convergent evolution in host defence. Similar signals of convergent evolution in the TLR1/2/6/10 cluster were recently reported between European and Rroma populations living in the same geographic area^25^. It has been proposed that these similar effects on different populations were exerted by plague^25^. Confirming this hypothesis, we see that most populations have suffered a strong selective pressure in the same direction in genes related to host defence against bacteria and malaria, leading to smaller inter-population genetic distances (Figs. 4,5). No such strong effects were present when genes important for antifungal host defence mechanisms were assessed (Fig. 6). This might be expected considering that life-threatening fungal infections occur mainly in immunocompromised settings due to either invasive medical procedures or HIV infections, both conditions not encountered in early history.

## Conclusions

In this work, we analyse genotyping data for almost 140,000 SNPs in nine East African populations from Sudan, South Sudan and Ethiopia. Our main results add new and interesting features to the North East African genetic complexity, with new populations that define a genetic component in southern Nilo-Saharan speakers that cannot be related to a North-African or other sub-Saharan components. These populations should be included in further population genetics and epidemiological studies to have a representative sample of the genetic diversity of the region of East Africa. Moreover, a functional analysis shows similar genetics signals related to genes involved in antimalarial and antibacterial immune response. These findings suggest convergent evolution of the immune system of various ethnic groups in East Africa due to the major common selective pressures attributable to parasitic and bacterial infections acting on these populations.

## Materials and Methods

### Samples

**Figure 1:**
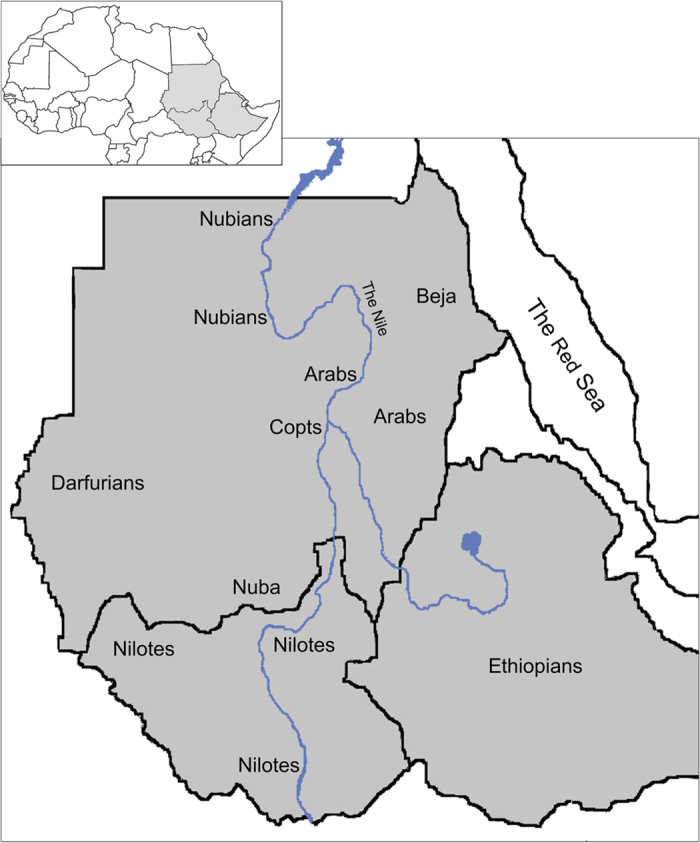
Map of studied region in East Africa showing Sudan, South Sudan and Ethiopia, and the approximate locations of eight populations genotyped in this study. Nomadic Fulani are not shown in the map due to their wide distribution in the west, central and east of the Sudan. The inset in the top shows the locations of Sudan, South Sudan and Ethiopia in East Africa. Modified from d-maps.com.

**Figure 2:**
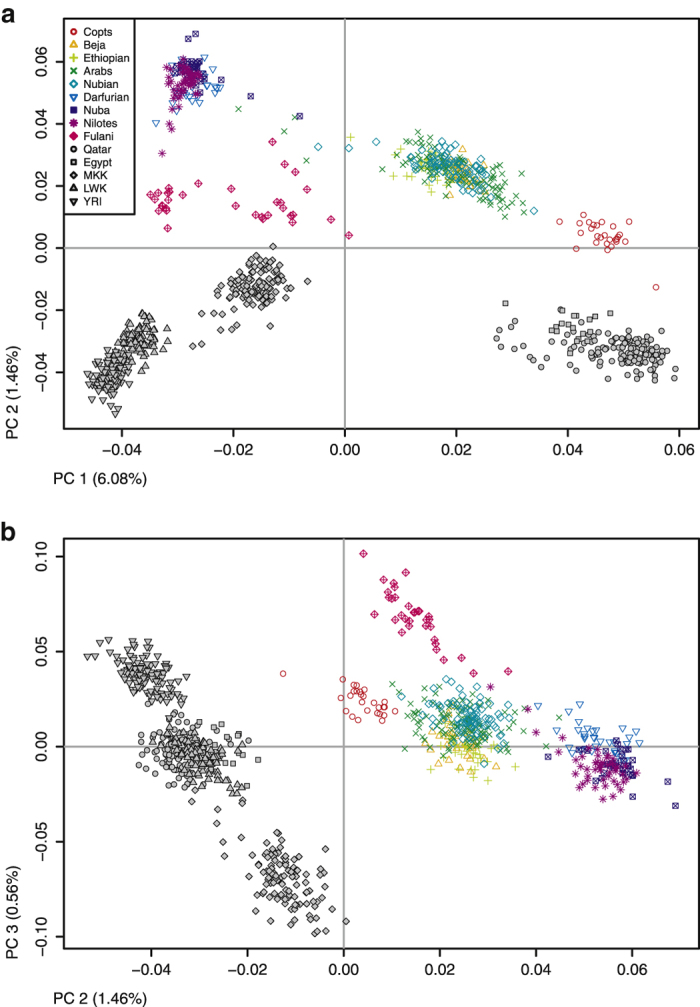
Principal component analysis of the populations from the Sudanese region in the context of the African continent. Plot shows **a**) PC1 and PC2 and **b**) PC2 and PC3 and the variation explained by them. Sudanese populations cluster in four groups according to their geographic location, with PC1 representing a north-east to south-west axis in East Africa. Populations not genotyped in this study are shown with grey filled symbols. MKK = Maasai from Kinyawa, Kenya; LWK = Luhya from Webuye, Kenya; YRI = Yoruba from Ibadan, Nigeria.

**Figure 3:**
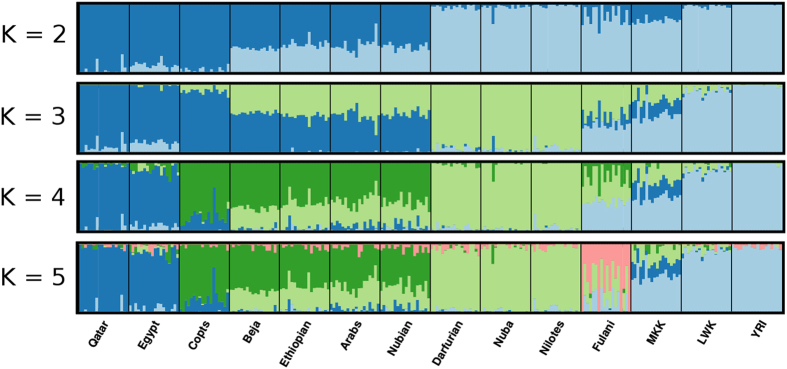
ADMIXTURE results for the 14 populations. A random subset of 18 individuals from each population was selected to avoid sample size bias. Columns represent individuals, where the size of each colour segment represents the proportion of ancestry from each cluster. Although k = 3 is the statistically supported model, here we show the results from k = 2 through k = 5 as they explain several ancestral components: North African/Middle Eastern (dark blue), Sub-Saharan (light blue), Coptic (dark green), Nilo-Saharan (light green) and Fulani (pink). MKK = Maasai from Kinyawa, Kenya; LWK = Luhya from Webuye, Kenya; YRI = Yoruba from Ibadan, Nigeria.

**Figure 4:**
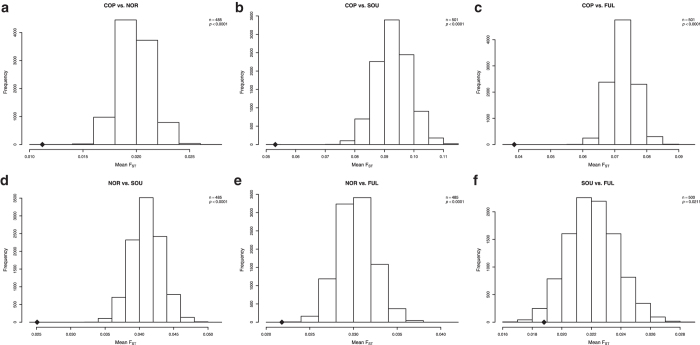
Genes associated with resistance/susceptibility to malaria. Sampling distribution of the sample mean pairwise F_ST_ between populations. Average F_ST_ value of genes associated with resistance/susceptibility to malaria (♦) is significantly lower than the mean F_ST_ score of the sampling distribution in all pairwise comparisons. COP = Copts; NOR = Beja, Ethiopians, Arabs and Nubians; SOU = Darfurians, Nuba and Nilotes; FUL = Fulani. The sampling distribution is drawn from the mean F_ST_ value of subsets of randomly selected genic SNPs with a sample size equal to the number of common SNPs between populations in the selected genes (n) and with similar MAF (10,000 permutations).

**Figure 5:**
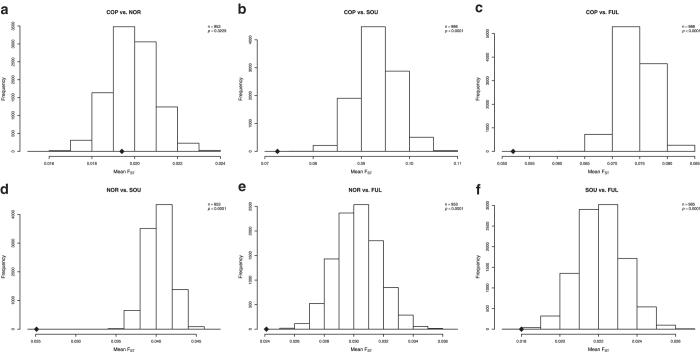
Anti-bacterial host defence related genes. Sampling distribution of the sample mean pairwise F_ST_ between populations. All pairwise comparisons, except COP vs. NOR, have an average F_ST_ value of anti-bacterial host defence related genes (♦) that is significantly lower than the mean F_ST_ score of the sampling distribution. COP = Copts; NOR = Beja, Ethiopians, Arabs and Nubians; SOU = Darfurians, Nuba and Nilotes; FUL = Fulani. The sampling distribution is drawn from the mean F_ST_ value of subsets of randomly selected genic SNPs with a sample size equal to the number of common SNPs between populations in the selected genes (n) and with similar MAF (10,000 permutations).

**Figure 6:**
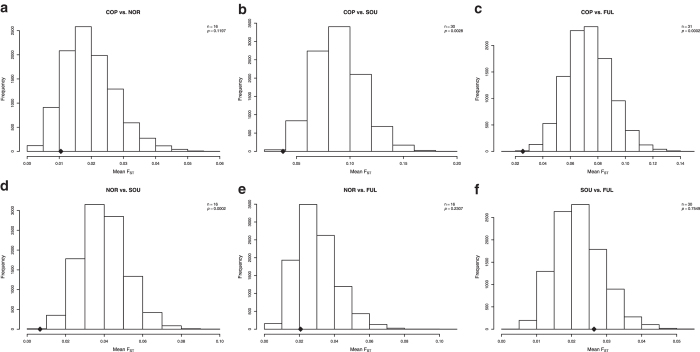
Anti-fungal host defence related genes. Sampling distribution of the sample mean pairwise F_ST_ between populations. Only three pairwise comparisons have an average F_ST_ value of anti-fungal host defence related genes (♦) that is significantly lower than the mean F_ST_ score of the sampling distribution: COP vs. SOU, COP vs. FUL, and NOR vs. SOU. COP = Copts; NOR = Beja, Ethiopians, Arabs and Nubians; SOU = Darfurians, Nuba and Nilotes; FUL = Fulani. The sampling distribution is drawn from the mean F_ST_ value of subsets of randomly selected genic SNPs with a sample size equal to the number of common SNPs between populations in the selected genes (n) and with similar MAF (10,000 permutations).

Saliva samples were collected from 500 individuals belonging to nine east African populations based on self-reported ethnicity. The samples used in the present research were collected and studied with ethical approval and informed consent. All experimental protocols were approved by the IRB of University of Medical Sciences and Technology in Khartoum and that of Universitat Pompeu Fabra (CEIC-IMAS; Comitè Ètic d’Investigació Clínica) in Barcelona and were carried out in accordance with the approved guidelines. The population samples from the Sudan belonged to: the Afro-Asiatic (Copts, n=40; Beja, n=40; and Arabs, n=120); the Nilo-Saharan (Nubians, n=80; Darfurians, n=50; and Nuba, n=21); and the Niger-Kordofanian linguistic families (Nuba, n=19). In addition to these populations, we also collected samples from neighbouring populations: Nilo-Saharan speaking Nilotes (n=50) from South Sudan, and Afro-Asiatic speaking Ethiopians (n=40) currently living in Khartoum (Sudan). Samples from Niger-Kordofanian speaking Fulani (n=40), a nomadic group that usually traverse Africa’s Sahel, were also analysed. These samples were genotyped on the Immunochip (Illumina Infinium single-nucleotide polymorphism microarray), a custom-made, high-density genotyping array containing 195,806 single-nucleotide polymorphisms (SNPs) and 718 small insertion-deletions^26^. Additional information about these populations is available in Table 1 (and Supplementary Table S1) and the sampling locations are shown in Fig. 1. Figure 1 was created by modifying 2 maps from d-maps.com website:

http://d-maps.com/carte.php?num_car=737&lang=en and http://d-maps.com/carte.php?num_car=740&lang=en, using Adobe Photoshop 7.0.1. software (Adobe Systems, San Jose, CA). Data are publicly available at the web page of JB (http://biologiaevolutiva.org/jbertranpetit/wp-content/uploads/2015/02/SudanImmunochip.zip) or under request.

Nine samples were identified as being second or third-degree relatives by identity by descent (IBD) matrix (IBD > 0.185) and were removed from the analyses. SNPs with a call rate below 99% and on sexual chromosomes were also removed. After these steps, a total of 143,602 SNPs and 447 samples remained: 27 Copts, 36 Beja, 39 Ethiopians, 112 Arabs, 79 Nubians, 35 Darfurians, 37 Nuba (18 Nilotic and 19 Niger-Kordofanian speakers), 49 Nilotes, and 33 Fulani. This data set is further referred to as the “140 K” data set.

For comparative studies, a Middle Eastern population (Qatar)^12^, a North African population (Egypt)^13^, and three sub-Saharan populations (Maasai, Luhya and Yoruba) from HapMap Phase 3^27^ were merged with the 140 K data set. These populations had 14,343 SNPs in common (“14 K” data set). See Supplementary Information for details.

### Population structure

To study the genetic relationships among East African ethno-linguistic groups, we used principal components analysis (PCA) as implemented in the Eigensoft package^28^.

Population differentiation was estimated using classical pairwise F_ST_ values^29^ for each pair of Sudanese populations for the 140 K data set. Then, we applied a Mantel test to study the correlation between geographic distance and genetic distance as measured by pairwise F_ST_ between populations. Mantel test was calculated using the R *ADE4* package^30^ with 10,000 permutations to estimate the statistical significance. Geographic distance was calculated as great-circle distances between populations. Nomadic Fulani were excluded from this last analysis due to their imprecise geographic distribution.

### Population admixture

Population admixture was analysed using ADMIXTURE^31^. This analysis identifies the genetic components of each group analysed and the ancestral clusters of the samples. It was run both on the 140 K data set of nine populations and on the 14 K data set of 14 populations (Sudan, South Sudan, Ethiopia, Egypt, Qatar and HapMap populations). To control for sample size differences, a random subset of 18 individuals was chosen for each population. Up to ten ancestral components (*k =* 2 through 10) were tested successively and the optimal value of *k* was estimated by ten-fold cross-validation. Clustering results were visualized with Distruct^32^.

To formally test whether admixture happened within the studied populations, and to measure its extend, we used the *three-population test* implemented in the ADMIXTOOLS software package^16^. This test is of the form f_3_ (X;A,B), where a negative value of the f_3_ statistic implies that population X (target population) was the result of an admixture event between the two ancestral populations of A and B (source populations). We tried every combination of source populations for each of our nine target populations and estimated the mixing coefficient (α) with Yoruba as the outgroup population. It is the proportion of the admixture of the target population given by the source population A, while 1 − α is the proportion given by the source population B. For each comparison we kept the results with a significantly negative value of the f_3_ statistic after multiple testing correction (Z-score <-4, p-value < 3.2×10^-5^).

### Infectious disease-related genes

To take advantage of the particular design of the array used, groups of functionally related genes were analysed to look for particular signals in a given population. Genes related with resistance/susceptibility to malaria^17^, and genes related to host defence against bacteria^18^ and fungi were selected for specific analyses (see Supplementary Table S5, S6 and S7 for the lists of genes). After controlling for population structure (see principal component and admixture analyses), populations were pooled in four groups to reduce the number of possible combinations: COP = Copts, NOR = North-East populations (Arabs, Beja, Ethiopians and Nubians), SOU = South-West populations (Darfurians, Nilotes and Nuba), and FUL = Fulani.

For every pair of groups (6 tests in total) we detected unusual allele frequency differentiation among them by F_ST_ statistics. For each SNP we computed the F_ST_ between each pair of groups or populations using the BioPerl module PopGen^33^. As the F_ST_ index is correlated with heterozygosity^34^,^35^,^36^, we compared the values with the ones observed at loci with similar MAF values. For that purpose, we divided the background SNP set into bins of similar number of SNPs and similar MAF values (0 − 0.04, 0.04 − 0.1, 0.1 − 0.2, 0.2 − 0.3, 0.3 − 0.4, 0.4 − 0.5). SNPs were assigned to a gene if they were up to 1 kb upstream or downstream of the transcription start site of that gene. SNPs were annotated using ANNOVAR^37^. For each pairwise comparison between populations, for each of the 3 functional categories of genes (malaria, bacterial, and fungal infections), the mean value of the F_ST_ score of those genes was compared to the sampling distribution of the average F_ST_ value of a subset of randomly selected genomic SNPs with the same sample size and similar MAF values than those of the functional categories. P values were calculated using a permutation test (10.000 permutations).

## Additional Information

**How to cite this article**: Dobon, B. *et al*. The genetics of East African populations: a Nilo-Saharan component in the African genetic landscape. *Sci. Rep.* **5**, 9996; doi: 10.1038/srep09996 (2015).

## Supplementary Material

Supplementary Information Supplementary Figures 1-6

## Figures and Tables

**Table 1:** Sample information of the populations genotyped in the present study; N, number of samples remaining after quality control filtering.

Ethnic Group	N	Socio-economical Activities	Geographic Region	Linguistic Family	Linguistic Subfamily
Copts	27	Agriculturist	Khartoum	Afro-Asiatic	Ancient Egyptian
Beja	36	Pastoralist	East Sudan	Afro-Asiatic	Cushitic
Ethiopians	39	Agro-pastoralist	Ethiopia	Afro-Asiatic	Cushitic
					Semitic
Arabs	113	Agriculturist	North Sudan	Afro-Asiatic	Semitic
		Pastoralist	Central Sudan		
			East Sudan		
Nubians	79	Agriculturist	North Sudan	Nilo-Saharan	Eastern-Sudanic
Darfurians	35	Agriculturist	West Sudan	Nilo-Saharan	Eastern-Sudanic
					Fur
					Maban
					Saharan
Nilotes	49	Pastoralist	South Sudan (Country)	Nilo-Saharan	Central-Sudanic
					Eastern-Sudanic
Nuba	37	Agro-pastoralist	South Sudan	Niger-Kordofanian	Kordofanian
				Nilo-Saharan	Kadugli-Krongo
					Eastern-Sudanic
Fulani	33	Nomadic Pastoralist	Sahel	Niger-Kordofanian	Atlantic

**Table 2:** Three-population test. Here we show the combinations of source populations that give the most negative f_3_ statistic (Z-score < -4, p-value < 3.2×10^–5^) for each target population (α_L_ is the lower bond and α_U_ is the upper bound of α, where α is the admixture proportion by which the target population was formed from the ancestral population of source population 1).

Target	Source1	Source2	f_3_	Z-score	α_L_	α_U_
Beja	Copts	Darfurians	−0.017997	−22.767	0.641	0.642
Arabs	Copts	Nuba	−0.022826	−31.657	0.583	0.637
Ethiopians	Copts	Nuba	−0.021254	−28.893	0.555	0.641
Nubians	Copts	Nuba	−0.02071	−27.231	0.602	0.683
Fulani	Copts	Yoruba	−0.0111	−11.19	0.308	0.368

Yoruba was used as outgroup population to estimate α except in Fulani, where the outgroup population used was Luhya.
