# Supplementary material for: The genetics of East African populations: a Nilo-Saharan component in the African genetic landscape
Source: Sci Rep. 2015 May 28;5:9996. doi: 10.1038/srep09996 (PMC4446898; doi:10.1038/srep09996)
Supplement: Supplementary Information — Supplementary Figures 1-6 [file srep09996-s1.pdf]

The genetics of East African populations: a Nilo-Saharan component in the African genetic landscape

Begoña Dobon[1#], Hisham Y. Hassan[2,3#], Hafid Laayouni[1,4#], Pierre Luisi[1], Isis Ricaño-Ponce [5], Alexandra Zhernakova[5], Cisca Wijmenga[5], Hanan Tahir[6], David Comas[1], Mihai G. Netea[7,8*] and Jaume Bertranpetit[1*]

[1]Institut de Biologia Evolutiva (UPF-CSIC), Departament de Ciències Experimentals i de la Salut, Universitat Pompeu Fabra, Barcelona, Catalonia, Spain. [2]College of Medical Laboratory Sciences, University of Science and Technology, Omdurman, Sudan; and [3]Banoon ART and Cytogenetics Centre, Bahrain defense Force Hospital, Manama, Kingdom of Bahrain. [4]Departament de Genètica i de Microbiologia, Grup de Biologia Evolutiva (GBE), Universitat Autonòma de Barcelona, Bellaterra (Barcelona), Spain. [5]University of Groningen, University Medical Center Groningen, Department of Genetics, Groningen, The Netherlands. [6]Sudan Medical and Scientific Research Institute, University of Medical Sciences and Technology, Khartoum, Sudan. [7]Department of Internal Medicine and [8]Radboud Center for Infectious Diseases, Radboud University Nijmegen Medical Centre, Nijmegen, The Netherlands.

# Equal contribution

*Corresponding authors: Mihai G. Netea (mihai.netea@radboudumc.nl) and Jaume Bertranpetit (jaume.bertranpetit@upf.edu)

SUPPLEMENTARY INFORMATION

Includes Supplementary Figures S1-S12, Supplementary Tables S1-S8 and Supplementary Methods.

SUPPLEMENTARY FIGURES

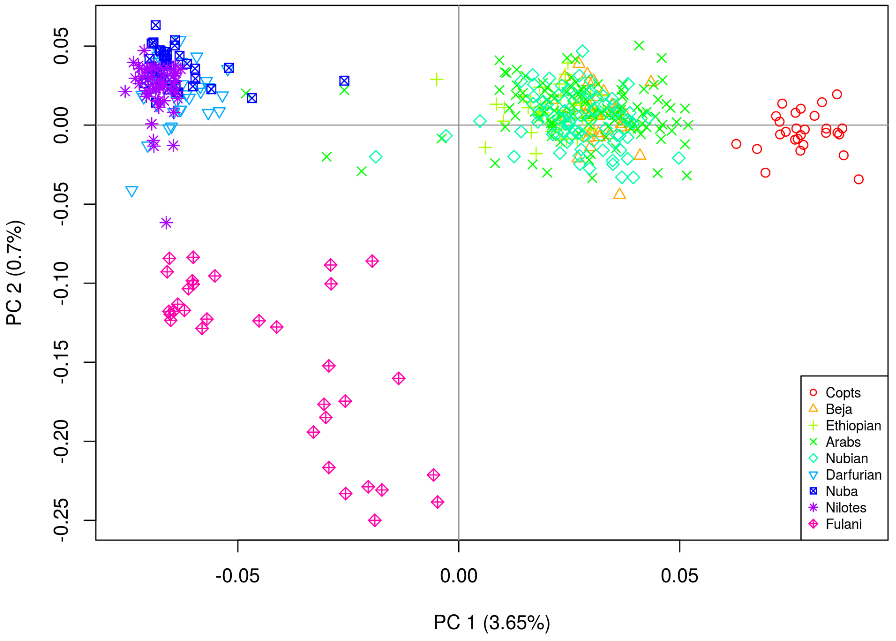

a)

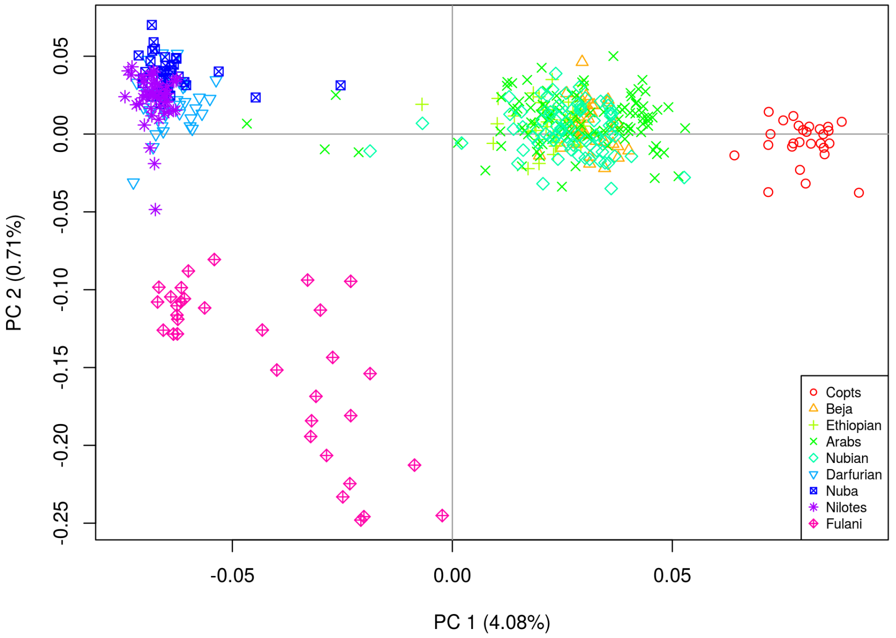

b)

Supplementary Fig. S 1. Principal component analysis of the new populations genotyped from the Sudanese region. Plot shows PC1 and PC2 and the variation explained by them using a) 143,602 markers and b) 14,343 markers (SNPs in common with the external populations in the 14K data set). PC1 separates the populations in a north-south gradient (Copts are originally from Egypt), whereas PC2 separates the nomadic Fulani from settled populations. The same structure between populations is recovered as in Fig. 2, even though the intra–population variation is higher.

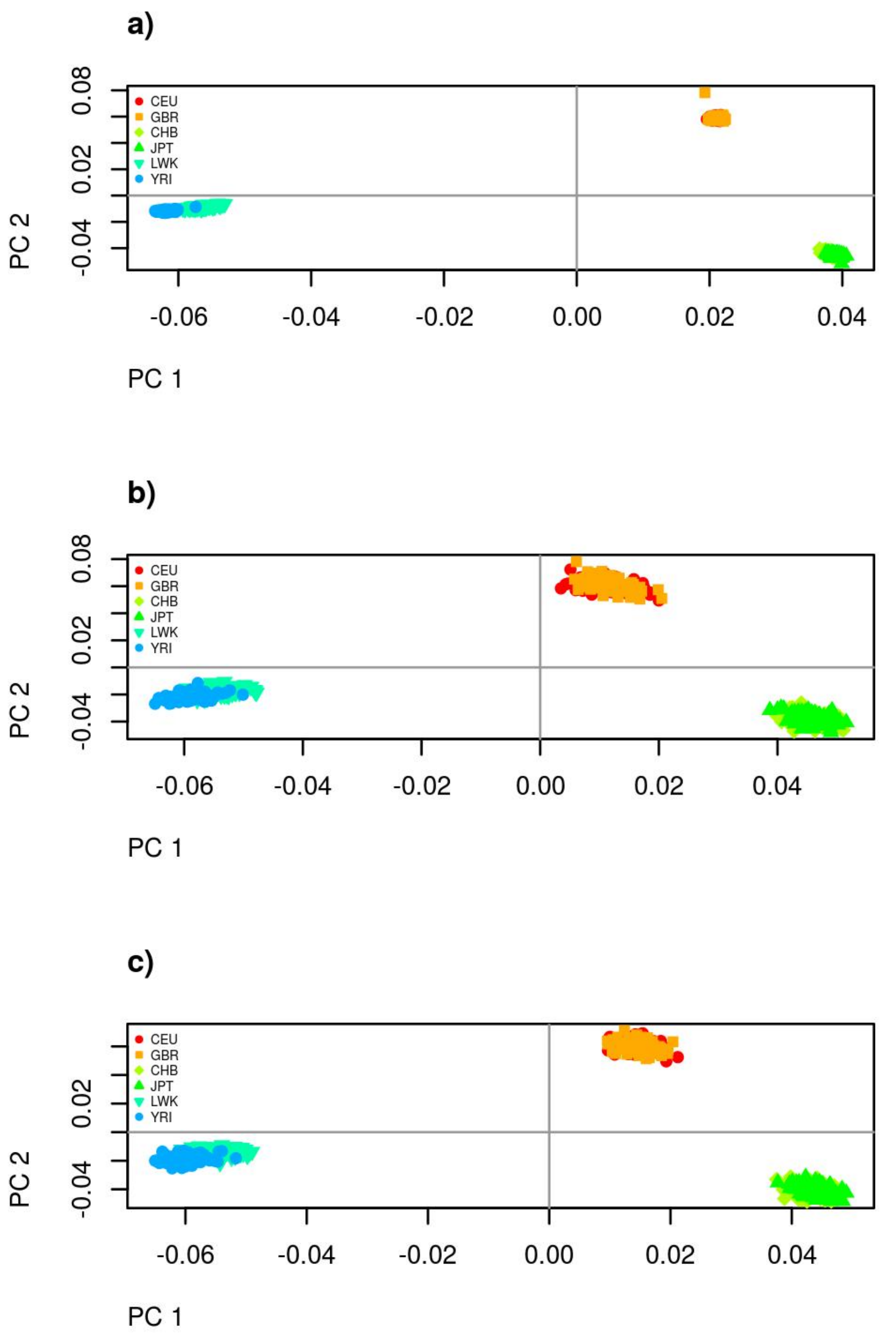

Supplementary Fig. S 2. Principal component analysis of six world–wide populations from 1000 Genomes Project using different number of SNPs. Plots show PC1 and PC2 using: a) 3M SNPs available from 1000 Genomes Project, b) 143,602 SNPs present in the Immunochip (140K data set), and c) 14,343 SNPs common between our newly genotyped populations and the reference populations we added to provide a continental context: Egyptian, Qatari, MKK, LWK and YRI samples (14K data set). Intra–population variability increases with lower number of SNPs, but the same population structure is recovered regardless of the number of SNPs. CEU = Utah residents with Northern and Western European ancestry; GBR = British in England and Scotland; CHB = Han Chinese in Bejing, China; JPT = Japanese in Tokyo, Japan (JPT); LWK = Luhya from Webuye, Kenya; and YRI = Yoruba from Ibadan, Nigeria.

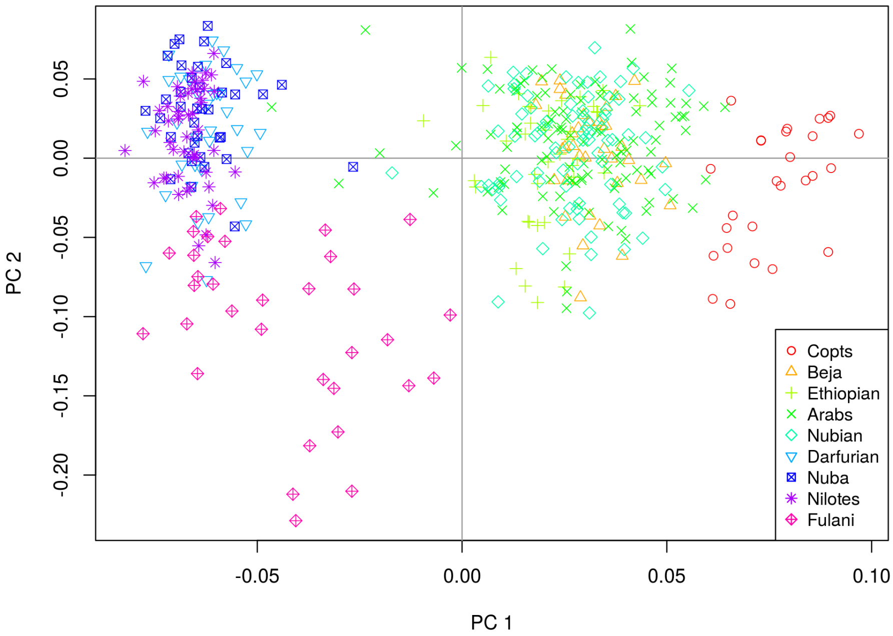
PC 2
0.05
0.00
-0.05
-0.10
-0.15
-0.20
-0.05
0.00
0.05
0.10
PC 1
Copts
Beja
Ethiopian
Arabs
Nubian
Darfurian
Nuba
Nilotes
Fulani

a)

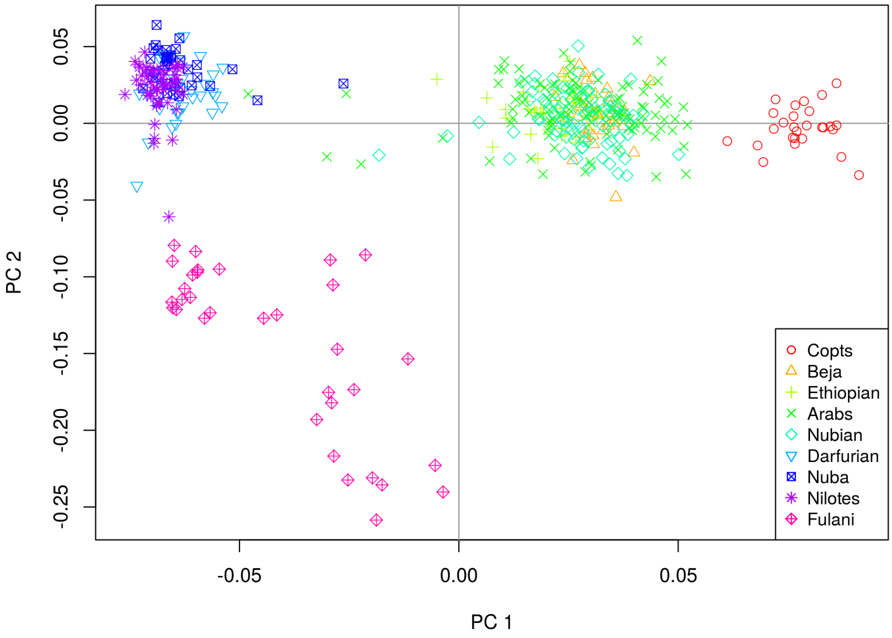
PC 2
0.05
0.00
-0.05
-0.10
-0.15
-0.20
-0.25
-0.05
0.00
0.05
PC 1
Copts
Beja
Ethiopian
Arabs
Nubian
Darfurian
Nuba
Nilotes
Fulani

b)

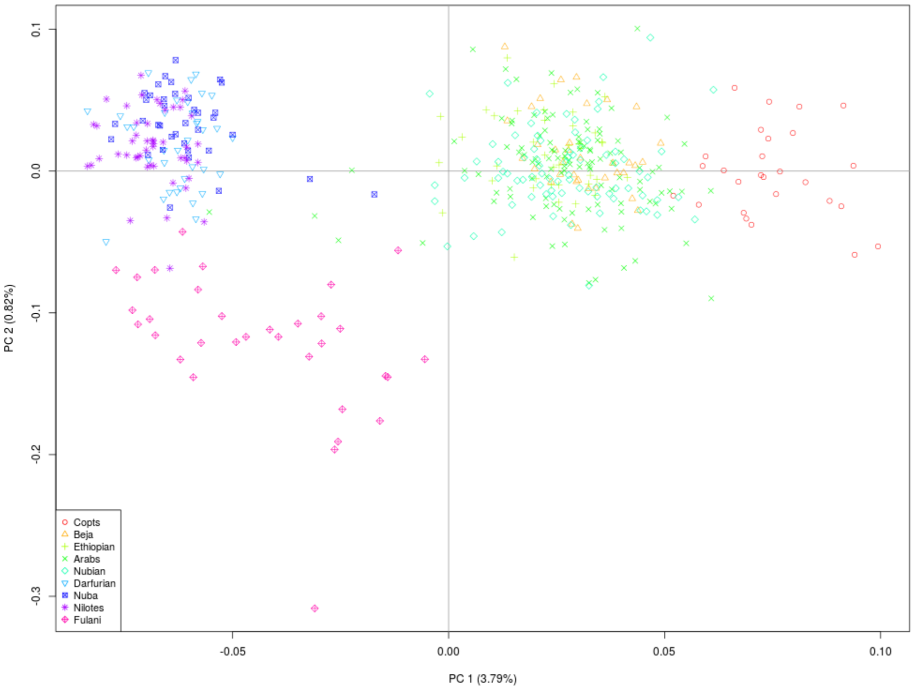

c)

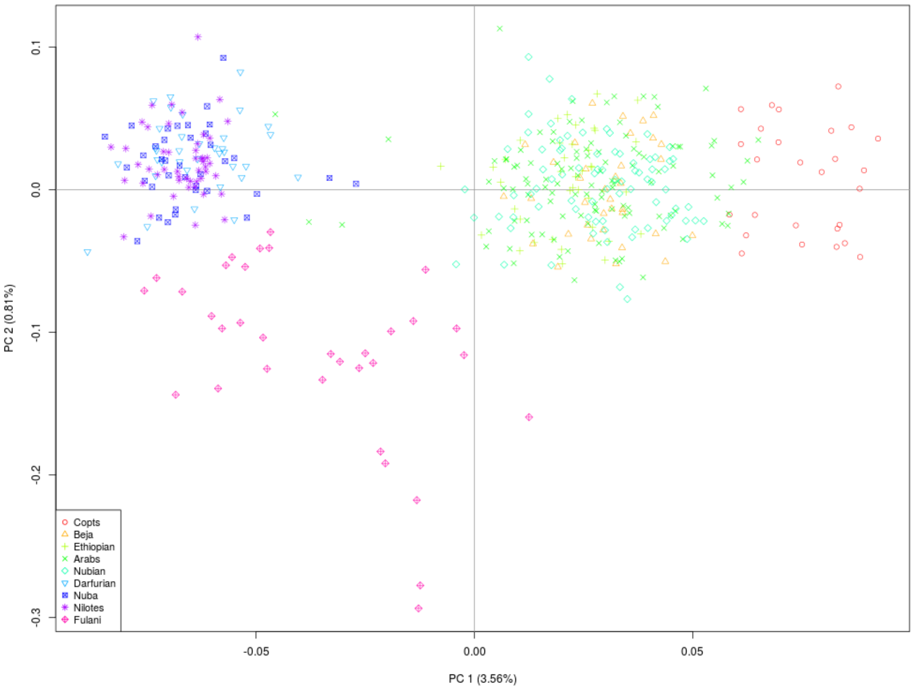

d)

Supplementary Fig. S 3. Principal component analysis of the new populations genotyped from the Sudanese region. Plot shows PC1 and PC2 using a) 9,221 genic SNPs and b) 133,724 intergenic SNPs (intergenic and intronics SNPs) genotyped in the Immunochip. c,d) We also performed PCA on ten random subsets of 10,000 intergenic SNPs to test whether the same population structure could be recovered with a equivalent sample size as the genic SNPs (shown two example plots). We observe a similar population structure as in Fig. 2, regardless of the subset of SNPs used.

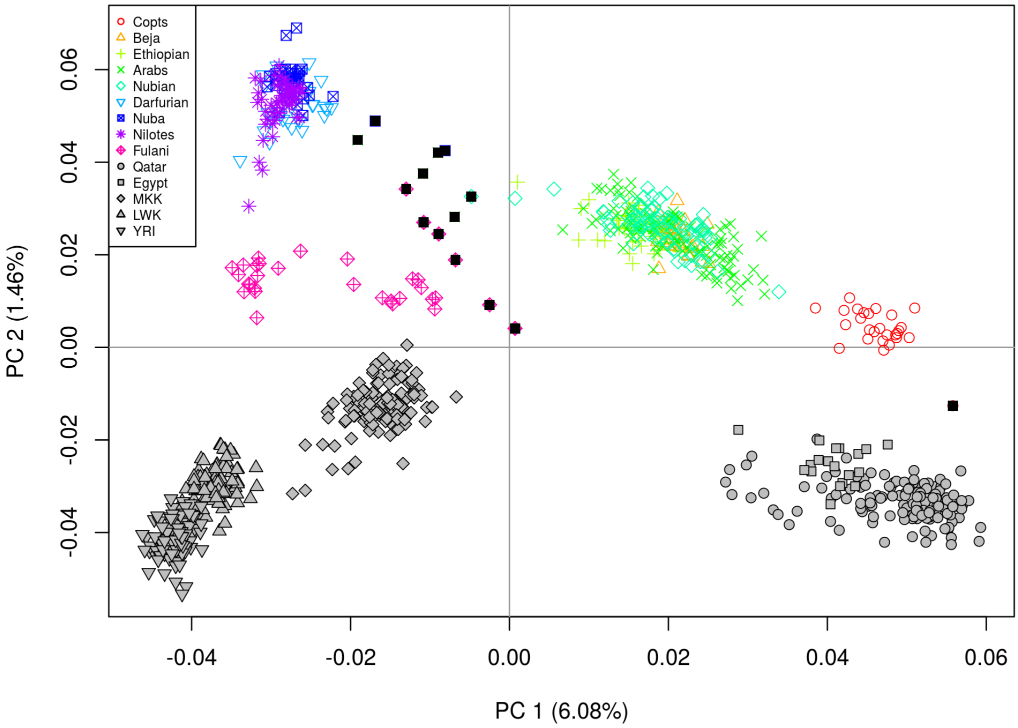

Supplementary Fig. S 4. Principal component analysis of the populations from the Sudanese region in the context of the African continent with 14 samples identified as outliers with respect to their populations of origin (■). The excluded individuals are: 1 Copt (211-5151), 1 Nubian (211-5042), 2 Nuba (211-4903, 211-4926), 4 Arabs (211-4969, 211-5187, 211-5212, 210-22416), and 6 Fulani (211-5208, 211-5281, 211-5215, 211-5238, 211-5198, 211-5230). MKK = Maasai from Kinyawa, Kenya; LWK = Luhya from Webuye, Kenya; YRI = Yoruba from Ibadan, Nigeria. Modified from main Figure 2a.

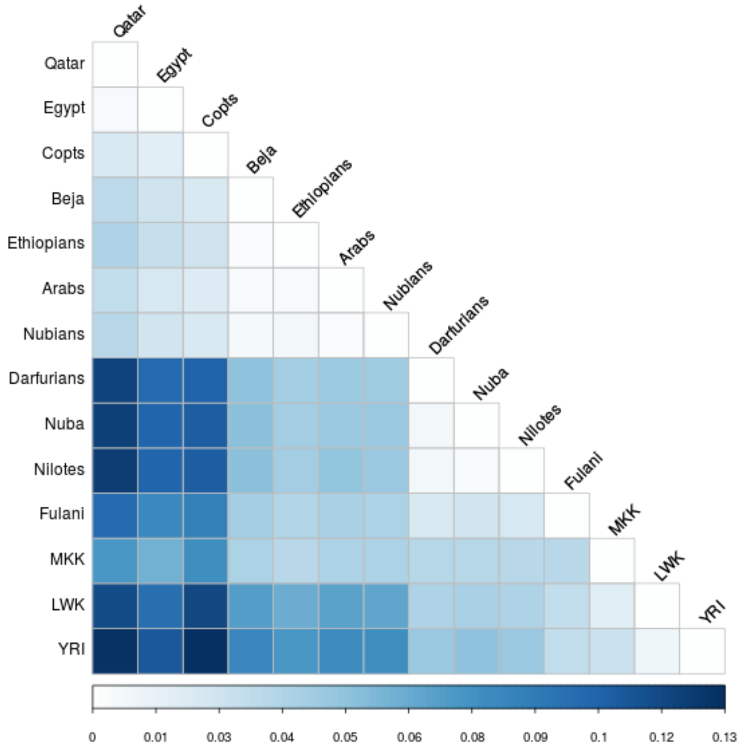

Supplementary Fig. S 5. Pairwise F_{ST} values between the 14 populations. Values of pairwise F_{ST} were colour coded to show the grades of genetic differentiation between populations. Geographically close populations have low genetic distances: Arabs, Beja, Etiophians and Nubians form one cluster (North-East) and Darfurian, Nilotes and Nuba another (South-West). MKK = Maasai from Kinyawa, Kenya; LWK = Luhya from Webuye, Kenya; YRI = Yoruba from Ibadan, Nigeria.

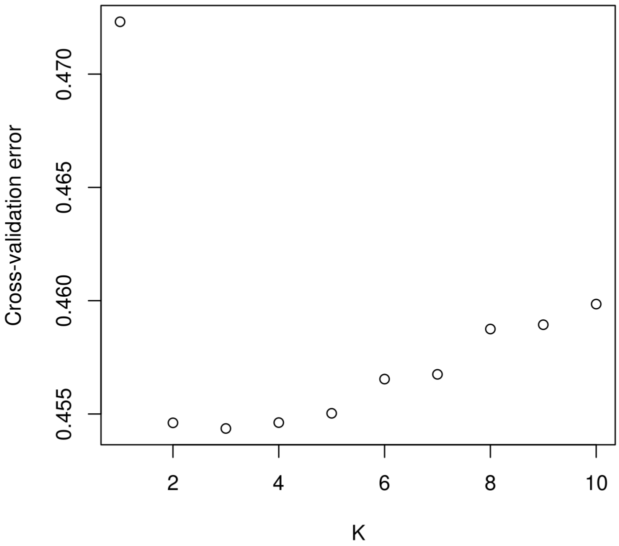

a)

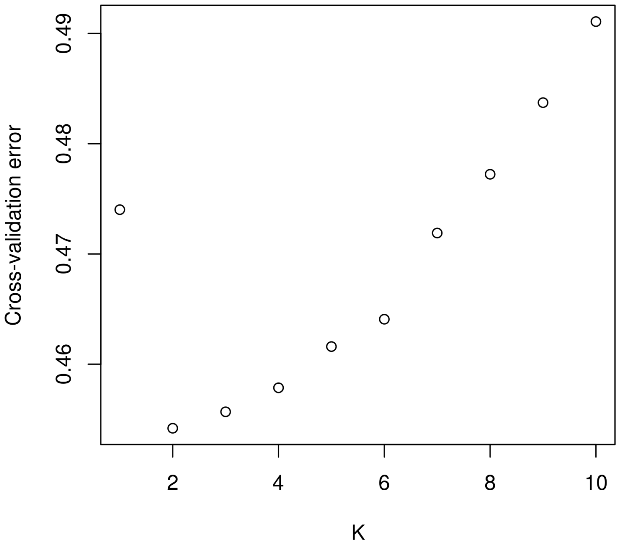

b)

Supplementary Fig. S 6. Cross-validation error estimates of the new nine genotyped populations for the ADMIXTURE analysis. Plots show standard error of the cross–validation error estimates for each of the 10 clusters tested (from k = 1 through k = 10) when a) all 447 individuals were analysed and b) a random subset of 25 individuals was chosen from each of the 9 populations to avoid sample size bias (225 individuals). When population sample size is taken into account the optimal number of ancestral clusters is two.

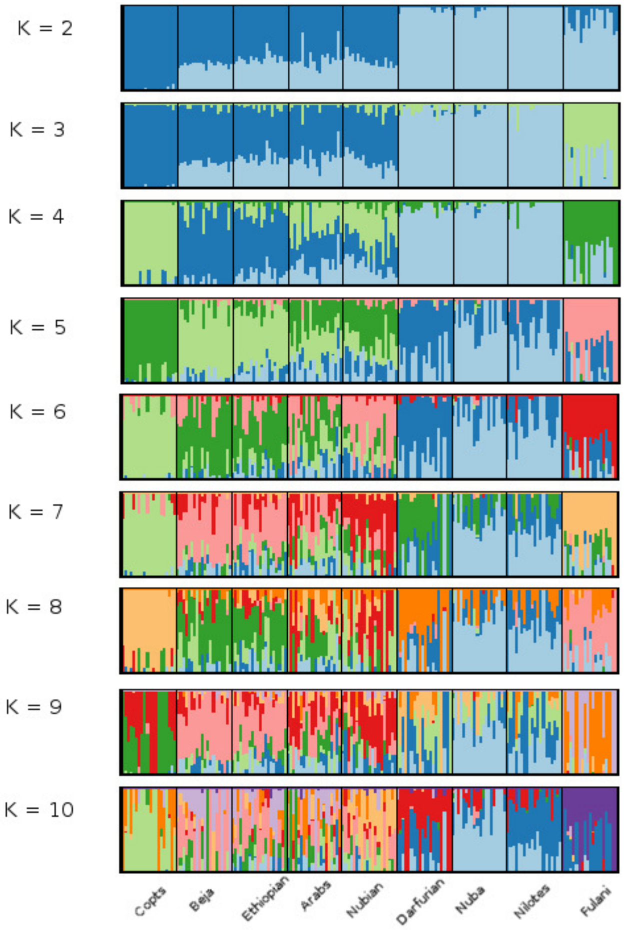

Supplementary Fig. S 7. ADMIXTURE results for $k = 2$ through $k = 10$ for the Sudanese populations. A random subset of 25 samples was extracted from each population to avoid sample size bias. Columns represent individuals, where the size of each colour segment represents the proportion of ancestry from each cluster. For the cross–validation error estimation, $k = 1$ was calculated but is not shown here. The statistically supported model is $k = 2$ (Supplementary Fig. 6b). A North–African ancestral component (dark blue) driven by the Coptic population is found in north-eastern populations (Arabs, Beja, Ethiopians and Nubian) along with a Nilo–Saharan ancestral component (light blue) characterised by south–western populations (Nuba, Nilotes and Darfurian). Nomadic Fulani are predominantly Nilo–Saharan, but at $k = 3$ show their own component (light green). Fulani individuals present different levels of admixture with a Nilo–Saharan component.

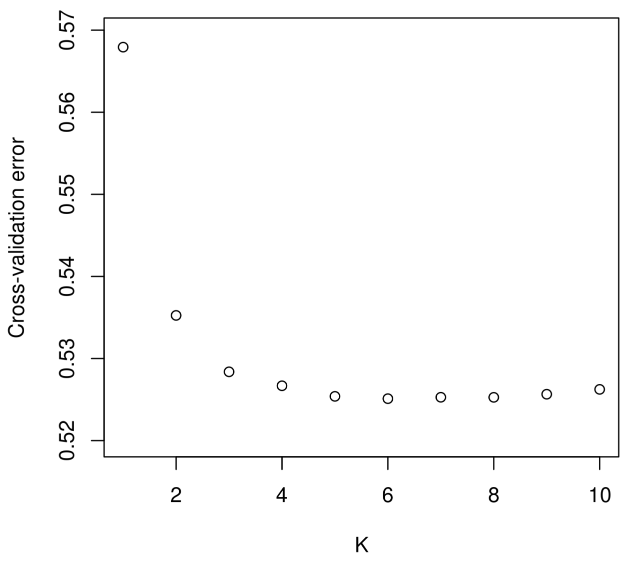

a)

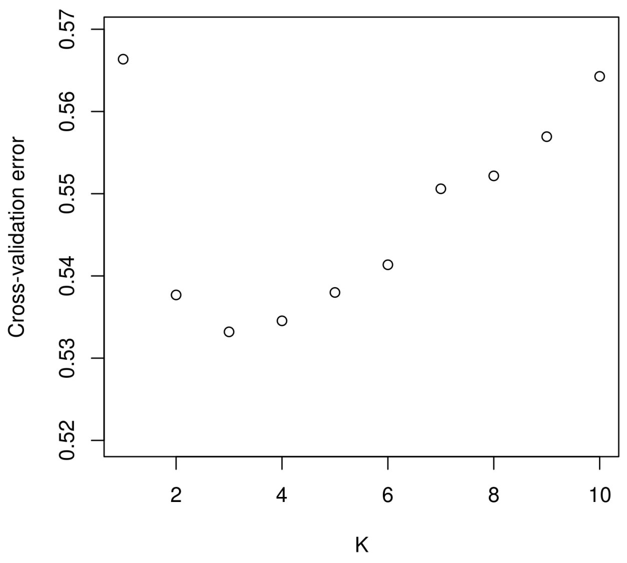

b)

Supplementary Fig. S 8. Cross-validation error estimates of the 14 populations for the ADMIXTURE analysis. Plots show standard error of the cross–validation error estimates for each of the 10 clusters tested (from $k = 1$ through $k = 10$) when a) all 921 individuals were analysed and b) a random subset of 18 individuals was chosen from each of the 14 populations to avoid sample size bias (252 individuals). When population sample size is taken into account the optimal number of ancestral clusters decreases by half (from six to three).

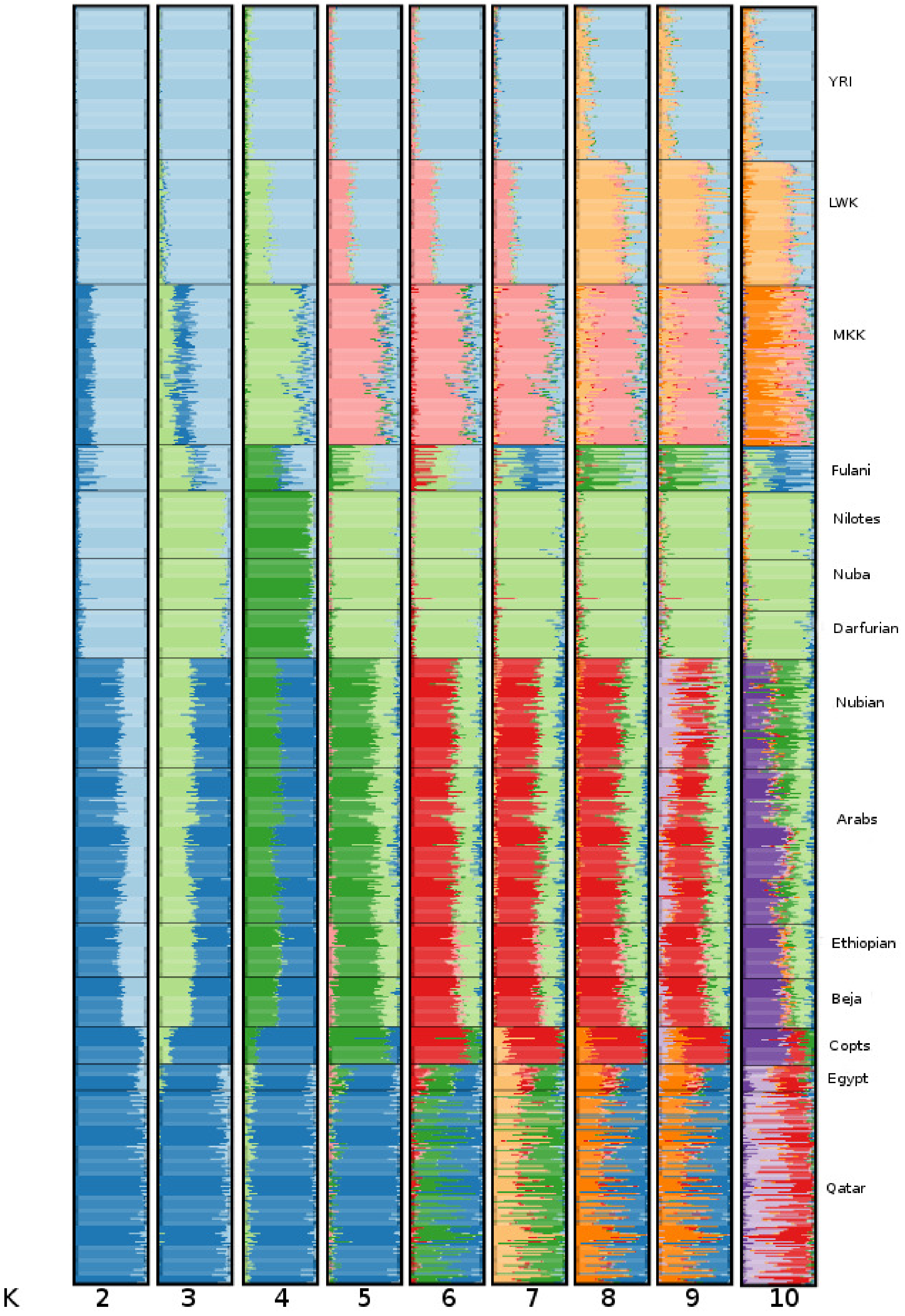

Supplementary Fig. S 9. ADMIXTURE results for $k = 2$ through $k = 10$ for the 14 populations using all 921 individuals. Columns represent individuals, where the size of each colour segment represents the proportion of ancestry from each cluster. For the cross-validation error estimation, $k = 1$ was calculated but is not shown here. The optimal number of clusters ranges from 5 to 7. MKK = Maasai from Kinyawa, Kenya; LWK = Luhya from Webuye, Kenya; YRI = Yoruba from Ibadan, Nigeria.

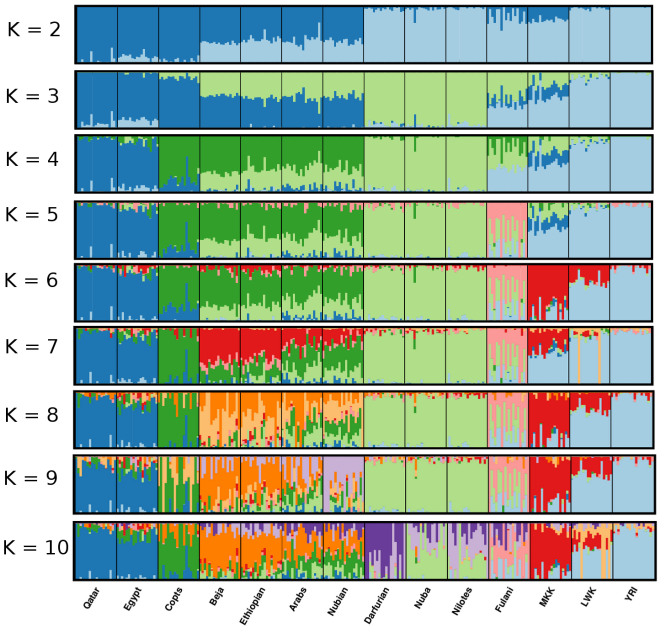

Supplementary Fig. S 10. ADMIXTURE results for $k = 2$ through $k = 10$ for populations from the Sudanese region in the context of other external populations. A random subset of 18 samples was extracted from each population to avoid sample size bias. Columns represent individuals, where the size of each colour segment represents the proportion of ancestry from each cluster. For the cross–validation error estimation, $k = 1$ was calculated but is not shown here. See main text and Fig. 3 for the interpretation of $k = 2$ through $k = 5$ of the ancestral components: North African/Middle Eastern (dark blue), Sub-Saharan (light blue), Coptic (dark green), Nilo–Saharan (light green) and Fulani (pink). MKK = Maasai from Kinyawa, Kenya; LWK = Luhya from Webuye, Kenya; YRI = Yoruba from Ibadan, Nigeria.

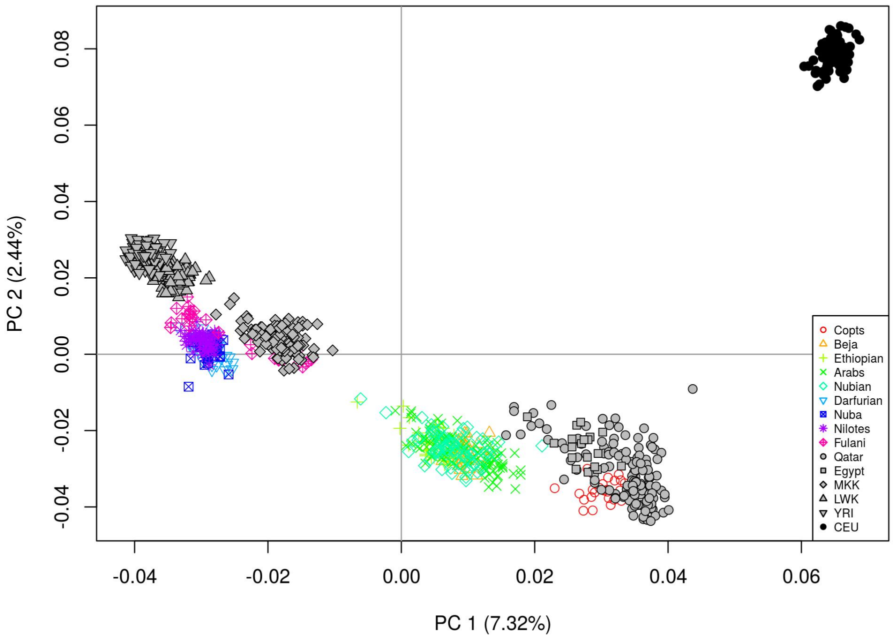

a)

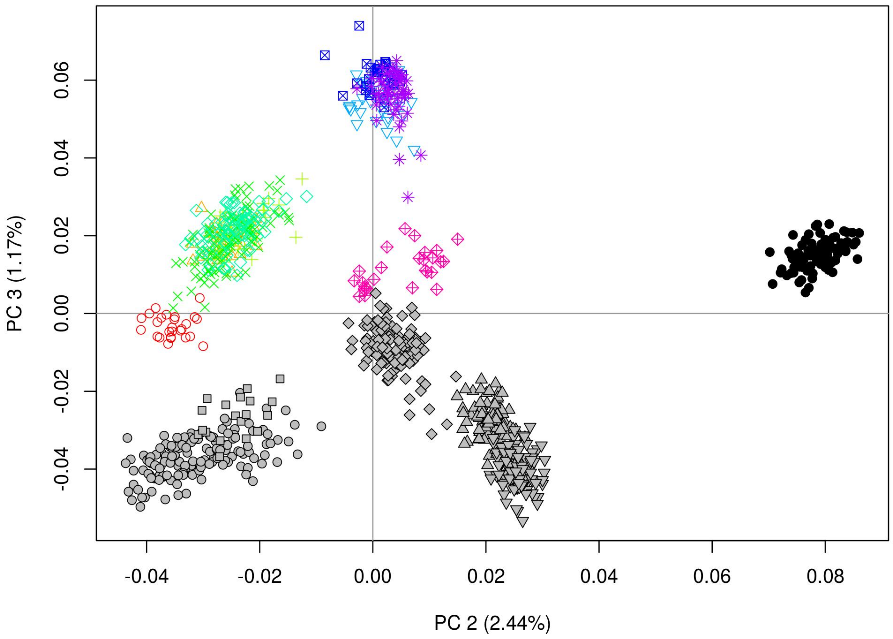

b)

Supplementary Fig. S 11. Principal component analysis of the populations from the Sudanese region in the context of the African continent with an European population added. Plot shows a) PC1 and PC2 and b) PC2 and PC3 and the variation explained by them using 13,713 markers. PC1 separates European, Middle East and North-African populations from Sub-Saharan populations. MKK = Maasai from Kinyawa, Kenya; LWK = Luhya from Webuye, Kenya; YRI = Yoruba from Ibadan, Nigeria; CEU = Utah Residents with Northern and Western European ancestry.

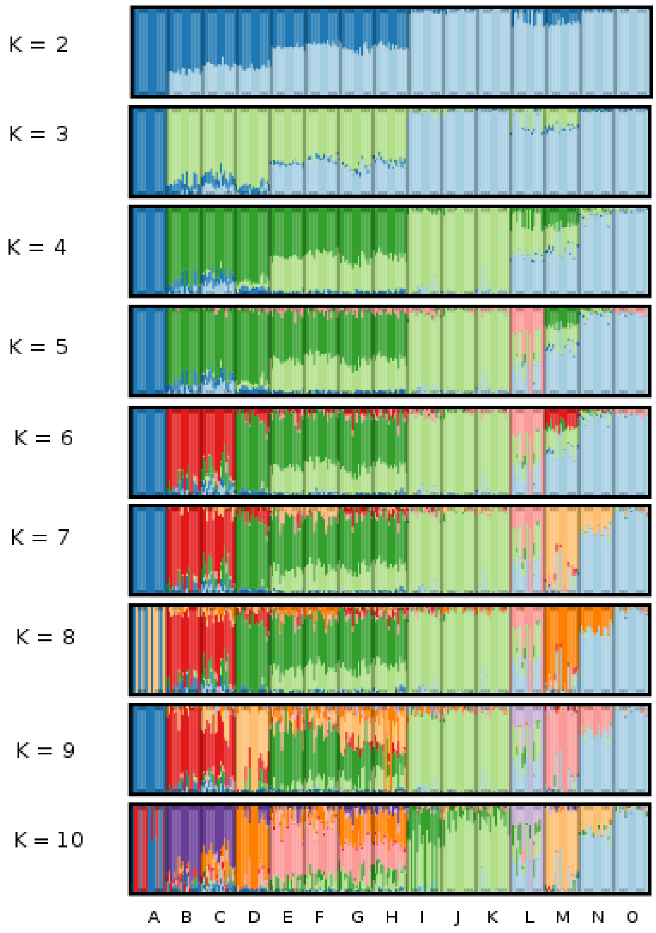

a)

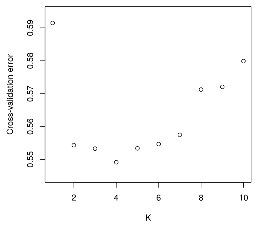

b)

Supplementary Fig. S 12. ADMIXTURE results for $k = 2$ through $k = 10$ for populations from the Sudanese region in the context of other external populations. a) A random subset of 18 samples was extracted from each population to avoid sample size bias. Columns represent individuals, where the size of each colour segment represents the proportion of ancestry from each cluster. b) For the cross–validation error estimation, $k = 1$ was calculated but is not shown here. A = Utah Residents with Northern and Western European ancestry; B = Qatar; C = Egypt; D = Copts; E = Beja, F = Ethiopians; G = Arabs; H = Nubians; I = Darfurians; J = Nuba; K = Nilotes; L = Fulani; M= Maasai from Kinyawa, Kenya (MKK) ; N = Luhya from Webuye, Kenya (LWK); O = Yoruba from Ibadan, Nigeria (YRI).

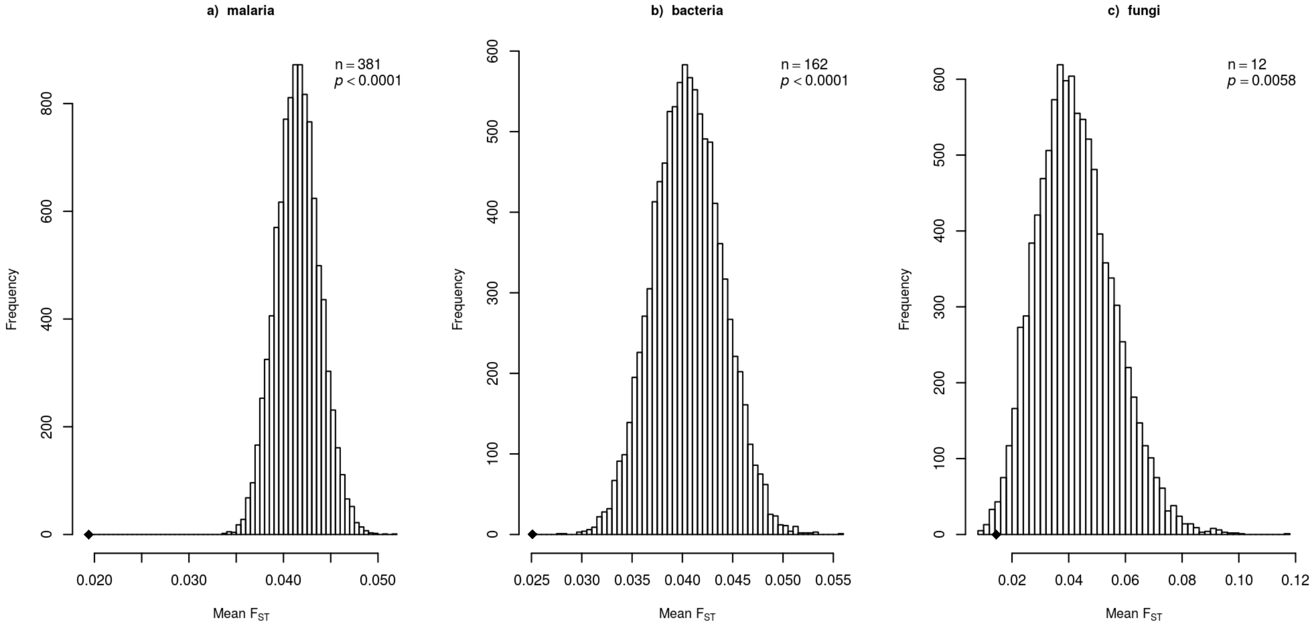

Supplementary Fig. S 13. Sampling distribution of the sample mean Global F_{ST} between Sudanese populations. The average F_{ST} value of genes associated with resistance/susceptibility to a) malaria, b) bacteria, and c) fungi (⧫) is significantly lower than the mean F_{ST} value of the sampling distribution. The sampling distribution is drawn from the mean F_{ST} value of subsets of randomly selected genic SNPs with a sample size equal to the number of common SNPs between populations in the selected genes (n) and with similar MAF (10,000 permutations).

SUPPLEMENTARY TABLES

Supplementary Table S1. Detailed sample information of the populations analysed in the present study, including sampling location and total number of individuals.

Ethnic Group	Ethnic Subgroup	Size	Socio-economical Activities	Country	Sampling Location	Coordinates	Linguistic Family	Linguistic Subfamily
Copts	Nagada	40	Agriculturist	Sudan	Khartoum	15N 32E	Afro-Asiatic	Ancient Egyptian
Beja	Bani Aamir	40	Pastoralist	Sudan	Sinkat	18N 36E	Afro-Asiatic	Cushitic
Ethiopian	Amhara	14	Agropastoralist	Ethiopia	Khartoum	15N 32E	Afro-Asiatic	Semitic
Ethiopian	Tigray	24	Agropastoralist	Ethiopia	Khartoum	15N 32E	Afro-Asiatic	Semitic
Ethiopian	Oromo	2	Agropastoralist	Ethiopia	Khartoum	15N 32E	Afro-Asiatic	Cushitic
Arabs	Shokrya	40	Pastoralist	Sudan	New Halfa	15N 35E	Afro-Asiatic	Semitic
Arabs	Shwaiga	40	Agriculturist	Sudan	Karima	18N 31E	Afro-Asiatic	Semitic
Arabs	Gaalien	40	Agriculturist	Sudan	Shendi	16N 33E	Afro-Asiatic	Semitic
Nubian	Halfawien	40	Agriculturist	Sudan	Wadi Halfa	21N 31E	Nilo-Saharan	Eastern-Sudanic
Nubian	Mahas	40	Agriculturist	Sudan	Kerma	19N 30E	Nilo-Saharan	Eastern-Sudanic
Darfurians	Fur	36	Agriculturist	Sudan	El-Fashir	13N 25E	Nilo-Saharan	Fur
Darfurians	Mima	1	Agriculturist	Sudan	El-Fashir	13N 25E	Nilo-Saharan	Fur
Darfurians	Tungor	1	Agriculturist	Sudan	El-Fashir	13N 25E	Nilo-Saharan	Eastern-Sudanic
Darfurians	Borgu	3	Agriculturist	Sudan	El-Fashir	13N 25E	Nilo-Saharan	Maban
Darfurians	Masalit	2	Agriculturist	Sudan	El-Fashir	13N 25E	Nilo-Saharan	Eastern-Sudanic
Darfurians	Karanga	3	Agriculturist	Sudan	El-Fashir	13N 25E	Nilo-Saharan	Eastern-Sudanic
Darfurians	Berti	1	Agriculturist	Sudan	El-Fashir	13N 25E	Nilo-Saharan	Saharan
Darfurians	Mararit	1	Agriculturist	Sudan	El-Fashir	13N 25E	Nilo-Saharan	Eastern-Sudanic
Darfurians	Tama	2	Agriculturist	Sudan	El-Fashir	13N 25E	Nilo-Saharan	Eastern-Sudanic
Nuba	Moro	10	Agropastoralist	Sudan	Kadugli	11N 29E	Niger-Kordofanian	Kordofanian
Nuba	Tuna	4	Agropastoralist	Sudan	Kadugli	11N 29E	Nilo-Saharan	Kadugli-Krongo
Nuba	Abu-Sinun	7	Agropastoralist	Sudan	Kadugli	11N 29E	Nilo-Saharan	Kadugli-Krongo
Nuba	Tagale	1	Agropastoralist	Sudan	Kadugli	11N 29E	Nilo-Saharan	Eastern-Sudanic
Nuba	Fama	1	Agropastoralist	Sudan	Kadugli	11N 29E	Nilo-Saharan	Kadugli-Krongo
Nuba	Rashad	1	Agropastoralist	Sudan	Kadugli	11N 29E	Niger-Kordofanian	Kordofanian
Nuba	Daier	1	Agropastoralist	Sudan	Kadugli	11N 29E	Nilo-Saharan	Eastern-Sudanic
Nuba	Atoro	1	Agropastoralist	Sudan	Kadugli	11N 29E	Niger-Kordofanian	Kordofanian
Nuba	Miri	1	Agropastoralist	Sudan	Kadugli	11N 29E	Nilo-Saharan	Kadugli-Krongo
Nuba	Kawalib	3	Agropastoralist	Sudan	Kadugli	11N 29E	Niger-Kordofanian	Kordofanian
Nuba	Temein/Aronga	3	Agropastoralist	Sudan	Kadugli	11N 29E	Nilo-Saharan	Eastern-Sudanic
Nuba	Dameek	1	Agropastoralist	Sudan	Kadugli	11N 29E	Nilo-Saharan	Kadugli-Krongo
Nuba	Karko	1	Agropastoralist	Sudan	Kadugli	11N 29E	Nilo-Saharan	Eastern-Sudanic

Ethnic Group	Ethnic Subgroup	Size	Socio-economical Activities	Country	Sampling Location	Coordinates	Linguistic Family	Linguistic Subfamily
Nuba	Julud	1	Agropastoralist	Sudan	Kadugli	11N 29E	Niger-Kordofanian	Kordofanian
Nuba	Shawabna	1	Agropastoralist	Sudan	Kadugli	11N 29E	Niger-Kordofanian	Kordofanian
Nuba	Tulishi	1	Agropastoralist	Sudan	Kadugli	11N 29E	Nilo-Saharan	Kadugli-Krongo
Nuba	Haiban	1	Agropastoralist	Sudan	Kadugli	11N 29E	Niger-Kordofanian	Kordofanian
Nuba	Tira Dagig	1	Agropastoralist	Sudan	Kadugli	11N 29E	Niger-Kordofanian	Kordofanian
Nilotes	Dinka	17	Pastoralist	South Sudan	Juba	4N 31E	Nilo-Saharan	Eastern-Sudanic
Nilotes	Baria	3	Pastoralist	South Sudan	Juba	4N 31E	Nilo-Saharan	Eastern-Sudanic
Nilotes	Balanda	1	Pastoralist	South Sudan	Juba	4N 31E	Nilo-Saharan	Eastern-Sudanic
Nilotes	Moru	1	Pastoralist	South Sudan	Juba	4N 31E	Nilo-Saharan	Central-Sudanic
Nilotes	Nuer	18	Pastoralist	South Sudan	Juba	4N 31E	Nilo-Saharan	Eastern-Sudanic
Nilotes	Kakwa	1	Pastoralist	South Sudan	Juba	4N 31E	Nilo-Saharan	Eastern-Sudanic
Nilotes	Shilluk	5	Pastoralist	South Sudan	Juba	4N 31E	Nilo-Saharan	Eastern-Sudanic
Nilotes	Anyuak	1	Pastoralist	South Sudan	Juba	4N 31E	Nilo-Saharan	Eastern-Sudanic
Nilotes	Tennet	1	Pastoralist	South Sudan	Juba	4N 31E	Nilo-Saharan	Eastern-Sudanic
Nilotes	Mundari	1	Pastoralist	South Sudan	Juba	4N 31E	Nilo-Saharan	Eastern-Sudanic
Fulani	–	40	Nomadic Pastoralist	Sahel	–	–	Niger-Kordofanian	Atlantic

Supplementary Table S2. Pairwise F_{ST} comparisons among the Sudanese ethno-linguistic groups and neighbouring populations.

	**Copts**	**Beja**	**Ethiopians**	**Arabs**	**Nubians**	**Darfurians**	**Nuba**	**Nilotes**
**Beja**	0.021							
**Ethiopians**	0.026	0.003						
**Arabs**	0.019	0.004	0.005					
**Nubians**	0.021	0.006	0.007	0.003				
**Darfurians**	0.104	0.052	0.044	0.047	0.046			
**Nuba**	0.106	0.054	0.045	0.049	0.048	0.007		
**Nilotes**	0.107	0.054	0.045	0.051	0.049	0.007	0.005	
**Fulani**	0.088	0.045	0.038	0.042	0.04	0.021	0.025	0.022

Supplementary Table S3. Three–population test with Yoruba as outgroup to estimate mixing proportions. Here we show all combinations of source populations that give a negative f_3 statistic (p–value < 3.2×10^{-5}) and the estimate mixing proportions are within bounds (α_L is the lower bond and α_U is the upper bound of α, where α is the admixture proportion by which was formed the target population from the ancestral population of source population 1). The pairs with the most negative results are summarized in Table 2 in the main text.

**Target**	**Source1**	**Source2**	f_3	**Z-score**	α_L	α_U
Beja	Fulani	Qatar	-0.010807	-12.874	0.102	0.404
Beja	Darfurians	Copts	-0.017997	-22.767	0.358	0.359
Beja	Nuba	Copts	-0.017329	-22.084	0.249	0.37
Beja	Nilotes	Copts	-0.01793	-21.981	0.314	0.361
Ethiopians	Fulani	Qatar	-0.013213	-16.313	0.196	0.477
Ethiopians	Darfurians	Copts	-0.021364	-28.487	0.339	0.431
Ethiopians	Nuba	Copts	-0.021254	-28.893	0.359	0.445
Ethiopians	Nilotes	Copts	-0.021274	-28.085	0.343	0.434
Ethiopians	Darfurians	Beja	-0.003714	-5.716	0.102	0.15
Ethiopians	Nuba	Beja	-0.004269	-6.537	0.11	0.158
Ethiopians	Nilotes	Beja	-0.003691	-5.576	0.104	0.152
Arabs	Fulani	Qatar	-0.014746	-19.195	0.211	0.446
Arabs	Nuba	Copts	-0.022826	-31.657	0.363	0.417
Arabs	Nilotes	Copts	-0.022784	-30.629	0.348	0.407
Arabs	Darfurians	Beja	-0.002611	-4.252	0.128	0.16
Arabs	Nuba	Beja	-0.002693	-4.254	0.112	0.169
Nubians	Fulani	Qatar	-0.013878	-17.824	0.169	0.431
Nubians	Luhya	Qatar	-0.007696	-5.193	0.345	0.356
Nubians	Darfurians	Copts	-0.020759	-26.402	0.304	0.385
Nubians	Nuba	Copts	-0.02071	-27.231	0.317	0.398
Nubians	Nilotes	Copts	-0.020634	-27.197	0.304	0.388

Supplementary Table S4. Three–population test with Luya as outgroup to estimate mixing proportions. Here we show all combinations of source populations that give a negative f_3 statistic (p–value < 3.2×10^{-5}) and the estimate mixing proportions are within bounds (α_L is the lower bond and α_U is the upper bound of α, where α is the admixture proportion by which was formed the target population from the ancestral population of source population 1).

**Target**	**Source1**	**Source2**	**f_3**	**Z-score**	**α_L**	**α_U**
Beja	Copts	Darfurians	-0.018	-22.77	0.639	0.658
Beja	Copts	Maasai	-0.0093	-12.78	0.633	0.657
Beja	Copts	Nuba	-0.0173	-22.08	0.632	0.699
Beja	Copts	Yoruba	-0.0153	-17.09	0.725	0.852
Ethiopians	Beja	Darfurians	-0.0037	-5.72	0.848	0.899
Ethiopians	Beja	Fulani	-0.0027	-4.39	0.841	0.897
Ethiopians	Beja	Nilotes	-0.0037	-5.58	0.853	0.903
Ethiopians	Beja	Nuba	-0.0043	-6.54	0.843	0.894
Ethiopians	Beja	Yoruba	-0.0043	-5.84	0.912	0.946
Ethiopians	Copts	Darfurians	-0.0214	-28.49	0.568	0.667
Ethiopians	Copts	Maasai	-0.0118	-16.76	0.559	0.564
Ethiopians	Copts	Nilotes	-0.0213	-28.09	0.574	0.638
Ethiopians	Copts	Nuba	-0.0213	-28.89	0.559	0.655
Ethiopians	Copts	Yoruba	-0.0192	-21.63	0.67	0.796
Ethiopians	Qatar	Yoruba	-0.0098	-6.35	0.621	0.863
Arabs	Beja	Darfurians	-0.0026	-4.25	0.834	0.892
Arabs	Beja	Nuba	-0.0027	-4.25	0.829	0.885
Arabs	Copts	Nuba	-0.0228	-31.66	0.586	0.619
Arabs	Copts	Yoruba	-0.0201	-23.85	0.69	0.789
Arabs	Qatar	Yoruba	-0.0111	-7.37	0.645	0.873
Nubians	Copts	Darfurians	-0.0208	-26.4	0.61	0.699
Nubians	Copts	Nilotes	-0.0206	-27.2	0.615	0.676
Nubians	Copts	Nuba	-0.0207	-27.23	0.602	0.688
Nubians	Copts	Yoruba	-0.0185	-20.78	0.702	0.81
Nubians	Qatar	Yoruba	-0.009	-5.85	0.655	0.903
Fulani	Copts	Yoruba	-0.0111	-11.19	0.308	0.368

Supplementary Table S5. List of genes related to resistance to malaria present in the Immunochip. Modified from Kwiatkowski[14].

Functional Classification	Genes
Common Erythrocyte Variants That Affect Resistance To Malaria	*SCN2A*
Cytoadherence	*CD36; ICAM1*
Immune Genes Reported to Be Associated with Different Malaria Phenotypes	*HLA–B; HLA–DR (A/B1/B5/B6); IL1B; IL12B; IL4; MBL2*

Supplementary Table S6. List of genes belonging to pathways related to antibacterial host defence present in the Immunochip. Modified from Casals *et al.*[15].

Functional Classification	Genes
Adaptors	*IRAK2; TIRAP; TRAF6*
Cytokines	*CCR2; CCR4; CCR6; CXCR6; IL10RA; IL10RB; IL12B; IL12RB1; IL12RB2; IL1B; IL21; IL21R; IL23R; IL4; IL4R; IL5RA; IL6; JAK3; LTBR; PTGDR; STAT3; TGFA; TGFB2; TGFBR2; TGFBR3*
Effectors	*C2; C3; C5; CFH; CLCA1; EGF; EGFR; ITGA2; ITGA8; ITGAL; MUC2; MUC7; NGFR; NOS1; NTRK1*
Modulators	*CD209; SOCS4; SOCS5*
Receptors	*BPI; CARD11; LBP; NOD1; TLR3*

Supplementary Table S7. List of genes belonging to fungi host defence present in the Immunochip. Provided by Mihai G. Netea.

Functional Classification	Genes
Adaptor	*SYK; STAT3*
Cytokines	*IL1B; IL6*
Receptor	*CLEC6A; MRC1; CLEC4E; CD209*

Supplementary Table S8. Summary statistics of SNPs of disease-related genes from African populations of 1000 Genomes Project compared to the portion of those SNPs genotyped in the Immunochip. N = number of SNPs analysed. S.d. = standard deviation.

	**SNPs from African populations**		**SNPs genotyped in the Immunochip**		**t-test**
**DISEASE**	Average F_{ST} ± sd	N	Average F_{ST} ± sd	N	
Malaria	0.0101 ± 0.0039	28499	0.0066 ± 0.0153	381	P = 0.2301
Bacteria	0.0075 ± 0.0033	61946	0.0088 ± 0.017	162	P = 0.1984
Fungi	0.0072 ± 0.0037	6558	0.0114 ± 0.0211	12	P = 0.369

SUPPLEMENTARY METHODS

Overview of the Immunochip

The Immunochip (Illumina Infinium single-nucleotide polymorphism microarray) was originally designed for deep replication of meta-genome-wide association studies (GWASs): 2.000 independent association signals for 11 immune-mediated diseases were included, based on early 1000 Genomes Pilot data (February 2010 release), mainly on European variants[1]. As result, 186 loci are dense covered with single-nucleotide polymorphisms (SNPs) while other genomic regions are not represented. This restricted the statistical methods that we could apply: methods based on linkage disequilibrium or haplotype information were discarded due to the heterogeneous SNP density. We focused our analysis in methods based on allele frequencies: pair-wise F_{ST}[2] and F-statistics[3]. Also, when working with genotyping arrays the problem of ascertainment bias should be addressed. To assess whether our inferences on population structure and population differentiation were robust to the peculiarities of the Immmunochip, we tested our assumptions using data from 1000 Genomes Project[4] with no ascertainment bias or from "neutral" Immmunochip SNPs.

Genotype Data and Quality Control

Samples were collected between June-September 2010 using Oragene.DNA saliva kit (Supplementary Table S1). Duplicated markers, and samples and markers with more than 2 % missing data rate were removed. There were 12 individuals with discordant sex

information. After manual revision and checking with the sample coordinator, sex codes based on SNP data were imputed in the original file and a new file with the revised gender assignments was created. Samples 211-4933 and 211-4934 had ambiguous sex codes and were removed. A sample without enough ethnic and linguistic information was removed (211-5344). Duplicated or/and related individuals were identified by identity by descent (IBD) matrix. Nine samples were identified as being second or third-degree relatives (IBD > 0.185) with respect to other individual and were removed: 4 Copts (211-5103, 211-5159, 211-5110, 211-5134), 4 Arabs (211-4947, 211-4898, 211-4921, 210-22425) and 1 Beja (211-5319).

Before, there were 176,562 markers and 459 samples. After these steps, 176,562 markers and 447 samples remained. A marker without a name was excluded from further analysis. SNPs on sexual chromosomes, with a minor allele frequency (MAF) less than 0.001 or that deviated from Hardy-Weinberg equilibrium (p-value < 1e-005) were removed.

A total of 143,602 SNPs and 447 samples remained: 27 Copts, 36 Beja, 39 Ethiopians, 112 Arabs, 79 Nubians, 35 Darfurians, 18 Nilo-Saharan Nuba, 19 Niger-Kordofanian Nuba, 49 Nilotes, and 33 Fulani. This data set is further referred to as the “140K” data set. We merged the SNPs genotyped on the Immunochip (140K data set), in the 116 Maasai from Kinyawa, Kenya (MKK), 90 Luhya from Webuye, Kenya (LWK) and 110 Yoruba from Ibadan, Nigeria (YRI) from HapMap 3[5]. Then, after merging them in the 19 Egyptian samples on the Affymetrix 6.0 platform[6], and in the 139 Qatari on the Affymetrix 500K platform[7], a total of 14,343 SNPs remained (“14K” data set). Two SNPs (rs10921143 and rs1627096) were removed as their alleles did not match. We updated genome positions from NCBI build 36 (UCSC hg 18) to NCBI build 37 (UCSC hg19) using the LiftOver utility from the University of California, Santa Cruz Genome Browser (http://genome.ucsc.edu/cgi-bin/hgLiftOver). Filtering and quality control was done using PLINK[8] and R[9].

Principal Component Analysis

Principal Component Analysis (PCA) was performed using Eigensoft (version 5.0.1) and all principal components plotted were significant (Tracy-Widom statistic p-value < 0.001)[10]. As the number of common markers between all platforms was relatively low (14,343 SNPs), we tested whether this number was enough to detect structure in African populations. We applied PCA on the 140K set using only the subset of common markers shared with the other genotyping platforms (14,343 SNPs). The same pattern between populations observed when all available SNPs are considered (Supplementary Fig. S1a, explained in main text) is recovered using less than 15,000 SNPs (Supplementary Fig. S1b). A minimum of 10,000 markers has been reported to be sufficient to identify population structure in African populations in a global context[11], and we show that less than 15,000 markers are enough to detect structure in a continental context.

Recovering Population Structure with the Immunochip

However, as mention before, the Immunochip does not have a representative selection of genome-wide SNPs. To assess whether the particular SNPs present in the Immunochip can recover population structure, we used data from the 1000 Genomes Project. We extracted a total of 545 individuals from world-wide populations: 85 Utah residents with Northern and Western European ancestry (CEU); 89 British in England and Scotland (GBR); 91 Han Chinese in Bejing, China (CHB); 94 Japanese in Tokyo, Japan (JPT); 97 Luhya in Webuye, Kenya (LWK); and 88 Yoruba in Ibadan, Nigeria (YRI)[4]. We applied a PCA using the different data sets of SNPs that we used for different analysis:

a) 3M SNPs available from 1000 Genomes Project data.
b) 140K data set: 143,602 SNPs genotyped in the Immunochip (after quality control) present in 1000 Genomes data.
c) 14K data set: 14,343 SNPs genotyped in the Immunochip (when our populations are merged with reference populations to provide continental context: Egyptian[5], Qatari[6], MKK, LWK and YRI samples[4]) present in 1000 Genomes data.

In Supplementary Fig. S2 we can see how the genetic structure between world-wide populations is maintained across the different data sets of SNPs used. Even though, the smallest data sets show higher intra-population variability, in our study we are able to separate between geographically close ethnic groups in our Sudanese populations.

We also evaluated the effect of ascertainment bias in the Immunochip using a subset of putative neutral SNPs (SNPs located in intergenic and intronic regions) by PCA. Supplementary Fig. S3 shows PC1 and PC2 using a) 9,221 genic SNPs and b) 133,724 intergenic SNPs (intergenic and intronics SNPs) genotyped in the Immunochip. We observe a similar population structure as in main Fig. 2, regardless of the subset of SNPs used. We also performed PCA on ten random subsets of 10,000 intergenic SNPs to test whether the same population structure could be recovered with an equivalent sample size as the genic SNPs (Supplementary Fig. S3c,d). We show no effect of ascertainment bias of the SNPs used on our conclusions.

Population differentiation

To emphasize population-specific characteristics, as analysed by genetic distances (pairwise F_{ST}), we excluded population outliers. After inspecting the PCA (main Fig. 2), 14

samples were identified as outliers with respect to their populations of origin. The excluded individuals are: 1 Copt (211-5151), 1 Nubian (211-5042), 2 Nuba (211-4903, 211-4926), 4 Arabs (211-4969, 211-5187, 211-5212, 210-22416), and 6 Fulani (211-5208, 211-5281, 211-5215, 211-5238, 211-5198, 211-5230). See Supplementary Fig. S4 to located the samples. Then, 433 individuals remained : 26 Copts, 36 Beja, 39 Ethiopians, 108 Arabs, 78 Nubians, 35 Darfurians, 18 Nilo-Saharan Nuba, 17 Niger-Kordofanian Nuba, 49 Nilotes, and 27 Fulani. Then, pairwise F_{ST} values for each pair of Sudanese populations was calculated (Supplementary Table S2, Supplementary Fig. S5)

Admixture Analysis

An unsupervised cluster algorithm, ADMIXTURE (version 1.23)[12] was applied both to the 140K and to 14K data sets. To test whether sample size difference would affect the results of ancestry estimation, a random subset of 25 (140K data set) or 18 (14K data set) individuals was chosen for each population. In each of these four data sets, ten ancestral clusters (k = 1 through 10) were tested successively. A 10-fold cross-validation procedure was applied to identify the value of k for which the model had the best predictive accuracy. Clustering results were visualized with Distruct[13].

In the 140K data set, when we use all individuals (447) the statistically supported model is k = 3 (Supplementary Fig. S6a). When we correct for sample size, the number of clusters that best fit our data decreases, being the best supported model k = 2 (Supplementary Fig. S6b). In the later case, populations from the Sudanese region differentiate in 3 groups: a group from one ancestral population formed by the Coptic population (dark blue), a second group from another ancestral population (light blue) formed by South-West populations (Nuba, Darfurians and Nilotes) and a third group of 70/30 mixed ancestry formed by North-

East populations (Beja, Arabs, Ethiopians and Nubians) (Supplementary Fig. S7). Fulani have mostly south-western ancestry, but they show their own component when the number of ancestral populations increases to k = 3 (light green). At k = 4, Beja and Ethiopian populations show a common component (dark blue). South-West populations form a homogeneous group until k = 5, when Darfurians (dark blue) start to differentiate from Nuba and Nilotes populations (light blue). At k = 6 Nubians separates from the rest. A higher number of ancestral populations do not help differentiate populations between themselves. This result is recovered when we use the 14K data set with the 14 populations (main text, Fig. 3).

When we run ADMIXTURE in the 14K data set using a random subset of 252 individuals (18 per population), the optimal number of ancestral clusters is 3 (explained in main text, Fig. 3, Supplementary Fig. S8b and S10). When we include all individuals in the analysis (921), the estimated number of ancestral populations doubles (k = 6) (Supplementary Fig. S8a). If we were to analyse the results with all individuals, we would see that at k = 4, Maasai would show their own component (light green) and Fulani would do it at k = 5 (pink) (Supplementary Fig. S9).

Ruling out recent European admixture

To eliminate the possibility of cryptic recent European admixture in our populations, we extracted 85 non-related individuals from the CEU population (Utah Residents with Northern and Western European ancestry) from the 1000 Genomes Project[4]. We merged them with the 14K data set and applied a PCA and ADMIXTURE analyses using the same procedure as before (see Principal Component and Admixture analyses).

In Supplementary Fig. S11, we can see how all African and Middle-East populations keep roughly the same relationships among themselves but cluster closer than in the previous PCA (see main Fig. 2). This is because the European population forms a separate cluster very different from the other African populations. The ADMIXTURE analysis reveals a new ancestral genetic component given by the European populations (dark blue at *k* = 2, Supplementary Fig. 12a). This component represents less than 5 % in the genetic composition of Middle-East and North-Africa populations. Now the best statistically supported model is *k* = 4, corresponding to the addition of the European population (Supplementary Fig. 12b). As the patterns of admixture between Sudanese populations do not change with the addition of a European genetic component, we ruled out the effect of possible recent European admixture in our populations.

F3 ancestry estimation

The three-population test is a formal test to detect admixture between populations implemented in the *qp3Pop* program from ADMIXTOOLS software package[3]. These tests are of the form f_3 (A; B, C), where a negative value of the f_3 statistic implies that population A (target population) comes from an admixture event between the two ancestral populations of B and C (source populations). The only situation when this test will not detect admixture is when the target population suffered a high-degree of population-specific drift after the admixture event.

The value of the mixing coefficient involving the first source population (α) can be estimated with *qpBound*[3]. This value should fall within reasonable boundaries: upper boundary of α should be higher than the lower boundary. To calculate the bonds of α we need an outgroup population that split from the ancestral population of A,B and C before populations A and B split. We used Yoruba as the outgroup population.

We tried every possible combination of source populations for each of our nine target populations. For each comparison we kept the results with a significantly negative value of the f_3 statistic (Z-score < -4, p-value < 3.2×10-5). All four target populations gave a significant result with an ancestral Middle East population (similar to the one that currently inhabits Qatar) and an ancestral West-Africa (similar to Fulani) as source populations (Table 2, Supplementary Table S3). This is a case when one of the source populations are themselves admixed, as are the Fulani. Beja appear as source population in Arabs and in Ethiopians with an ancestral South-West Sudanese population (Darfurians, Nuba or Nilotes) as the other source population. This could be because Beja and Ethiopians are very similar populations (F_{ST} = 0.003).

To confirm our results and the choice of Yoruba as the outgroup population, we also estimated the mixing proportions with Luhya instead of Yoruba (Supplementary Table S4). Then, the Yoruba population appears in all comparisons, giving a statistically significant value of f_3 and an α value within reasonable boundaries. This is an "outgroup case", where the ancestral population of Yoruba split from the ancestral population of A,B before they did. Despite of the outgroup population used, the comparisons giving the most negative values are the same, confirming that the choice of outgroup is not that relevant for these calculations[3].

Infectious disease-related genes

To assess whether the SNPs present in the Immunochip for genes related to infectious diseases are a random subset of all the SNPs of those genes, we compared the average F_{ST} values of two sets of SNPs. The first set is formed by all SNPs that belong to Immunochip genes related to resistance to bacteria, malaria or fungi and present in African populations of 1000 Genomes data[4]. The second set are SNPs that correspond to those present in the Immunochip, belonging to genes related to resistance to malaria, bacteria or fungi, and are also present in the 1000 Genomes data (see Suplementary Tables S5, S6, and S7). Set 2 is then formed by SNPs of our study and is obviously a subset of Set 1.

We extracted those SNPs from African populations (LWK and YRI) and compared the average F_{ST} value (mean ± standard deviation) of Set 1 and Set 2 using a t-test. We found that the average F_{ST} values of Immunochip SNPs of infectious disease-related genes are not statistically different from the average F_{ST} values of all SNPs of infectious disease-related genes of African populations from 1000 Genomes Project (Supplementary Table S8). Thus, the SNPs present in the Immunochip for those genes can be considered as a representative sample of all the SNPs in those genes.

We can take the same approach using global F_{ST} values instead of pairwise F_{ST} distances between our 4 sets of Sudanese populations. Then, we observe that globally genes related to resistance to bacteria, malaria or fungi have an average F_{ST} value that is statistically lower than the mean of the sampling distribution (Supplementary Fig. S13). However, as shown by the pairwise comparisons these results are driven by the similarities of some populations and not by all of them.

REFERENCES

1. Trynka, G. *et al.* Dense genotyping identifies and localizes multiple common and rare variant association signals in celiac disease. *Nat. Genet.* **43,** 1193–201 (2011).

2. Weir, B. S. & Hill, W. G. Estimating F-statistics. *Annu. Rev. Genet.* **36,** 721–750 (2002).

3. Patterson, N. *et al.* Ancient admixture in human history. *Genetics* **192,** 1065–93 (2012).

4. Abecasis, G. R. *et al.* An integrated map of genetic variation from 1,092 human genomes. *Nature* **491,** 56–65 (2012).

5. Altshuler, D. M., Gibbs, R. A. & Peltonen, L. T. I. H. 3 C. Integrating common and rare genetic variation in diverse human populations. *Nature* **467,** 52–58 (2010).

6. Henn, B. M. *et al.* Genomic ancestry of North Africans supports back-to-Africa migrations. *PLoS Genet.* **8,** e1002397 (2012).

7. Hunter-Zinck, H. *et al.* Population Genetic Structure of the People of Qatar. *Am. J. Hum. Genet.* **87,** 17–25 (2010).

8. Purcell, S. *et al.* PLINK: a tool set for whole-genome association and population-based linkage analyses. *Am. J. Hum. Genet.* **81,** 559–75 (2007).

9. R Core Team. *R: A Language and Environment for Statistical Computing*. (R Foundation for Statistical Computing, 2014).

10. Patterson, N., Price, A. L. & Reich, D. Population structure and eigenanalysis. *PLoS Genet.* **2,** e190 (2006).

11. Sikora, M., Laayouni, H., Calafell, F., Comas, D. & Bertranpetit, J. A genomic analysis identifies a novel component in the genetic structure of sub-Saharan African populations. *Eur. J. Hum. Genet.* **19,** 84–8 (2011).

12. Alexander, D. H. & Lange, K. Enhancements to the ADMIXTURE algorithm for individual ancestry estimation. *BMC Bioinformatics* **12,** 246 (2011).

13. Rosenberg, N. a. Distruct: a Program for the Graphical Display of Population Structure. *Mol. Ecol. Notes* **4,** 137–138 (2003).

14. Kwiatkowski, D. P. How malaria has affected the human genome and what human genetics can teach us about malaria. *Am. J. Hum. Genet.* 171–192 (2005).

15. Sikora, M., Ferrer-Admetlla, A., Mayor, A., Bertranpetit, J. & Casals, F. Evolutionary analysis of genes of two pathways involved in placental malaria infection. *Hum. Genet.* **123,** 343–57 (2008).
